# Efficacy of Drug-Coated Balloon Approaches for *de novo* Coronary Artery Diseases: A Bayesian Network Meta-Analysis

**DOI:** 10.3389/fcvm.2022.899701

**Published:** 2022-06-21

**Authors:** Peng-Yu Zhong, Ying Ma, Yao-Sheng Shang, Ying Niu, Nan Bai, Zhi-Lu Wang

**Affiliations:** ^1^Department of Cardiology, Nanchong Central Hospital, Nanchong, China; ^2^The First Clinical Medical College of Lanzhou University, Lanzhou, China; ^3^Department of Cardiology, The First Hospital of Lanzhou University, Lanzhou, China

**Keywords:** *de novo* coronary artery diseases, percutaneous coronary intervention, drug-eluting stents, drug-coated balloon, network meta-analysis

## Abstract

**Background and Objective:**

The *de novo* coronary lesions are the most common form of coronary artery disease, and stent implantation still is the main therapeutic strategy. This network meta-analysis aims to evaluate the efficacy of drug-coated balloons only (DCB only) and DCB combined with bare-metal stents (DCB+BMS) strategies vs. drug-eluting stents (DES) and BMS approaches in coronary artery *de novo* lesion.

**Method:**

PubMed, EMBASE, and Cochrane Library databases were retrieved to include the relevant randomized controlled trials that compared DCB approaches and stents implantation in patients with *de novo* coronary artery diseases. The primary outcome was major adverse cardiac events (MACE). The clinical outcomes included target lesion revascularization (TLR), all-cause death, and myocardial infarction. The angiographic outcomes consisted of in-segment late lumen loss (LLL) and binary restenosis. The odds ratio (OR) and 95% confidence intervals (95% CIs) for dichotomous data, and weighted mean differences for continuous data were calculated in the Bayesian network frame.

**Result:**

A total of 26 randomized controlled trials and 4,664 patients were included in this study. The DCB-only strategy was comparable with the efficacy of MACE, clinical outcomes, and binary restenosis compared with DES. In addition, this strategy can significantly reduce the in-segment LLL compared with the first-generation (MD −0.29, −0.49 to −0.12) and the second-generation DES (MD −0.15, −0.27 to −0.026). However, subgroup analysis suggested that DCB only was associated with higher in-segment LLL than DES (MD 0.33, 0.14 to 0.51) in patients with acute coronary syndrome. Compared with DES, the DCB+BMS strategy had a similar incidence of myocardial infarction and all-cause death, but a higher incidence of MACE, TLR, and angiographic outcomes. In addition, DCB+BMS was associated with a similar incidence of myocardial infarction and all-cause death than BMS, with a lower incidence of MACE, TLR, and angiographic outcomes.

**Conclusion:**

The DCB only is associated with similar efficacy and lower risk of LLL compared with DES. In addition, the DCB+BMS strategy is superior to BMS alone but inferior to DES (PROSPERO, CRD 42021257567).

**Systematic Review Registration:**

https://www.crd.york.ac.uk/PROSPERO/#recordDetails.

## Introduction

Coronary artery diseases are the most common type of cardiovascular disease and have become the major cause of cardiovascular death (1). The *de novo* coronary artery lesions refer to those that have not been treated with angioplasty or stenting, and stent implantation has become a standard strategy. Bare-metal stent (BMS) is the first-generation stent for coronary artery, which was applied in clinical practice in the 1980s. However, the risk of in-stent restenosis is high due to endothelial cell proliferation after BMS implantation. On the contrary, with the development of the platform, polymer, and anti-proliferative agents, drug-eluting stents (DES) have been widely applied in patients with coronary artery diseases. DES can significantly reduce the incidence of restenosis, and new-generation DES is associated with lower in-stent thrombosis rates than BMS during the first year (2, 3). However, patients undergoing percutaneous coronary intervention should accept 6–12 months dual antiplatelet therapy. The 2017 European Society of Cardiology guideline recommends 6 and 12 months dual antiplatelet therapy for patients with chronic coronary syndrome and acute coronary syndrome, respectively (4). Most importantly, stent implantation is an effective and safe strategy for the treatment of *de novo* coronary diseases, but there are still many limitations to be considered.

The drug-coated balloon (DCB) is an established therapy approach for in-stent restenosis after BMS or DES implantation, which is recommended by the guidelines of the European Society of Cardiology (Class I recommendation, level of evidence A) (5, 6). Meanwhile, DCB also is applied for the treatment of *de novo* coronary artery lesions. A meta-analysis of 14 randomized controlled trials suggested that DCB combined BMS (DCB+ BMS) strategy can significantly reduce the incidence of late lumen loss (LLL) and major adverse cardiac events (MACE) in patients with *de novo* coronary artery disease compared with BMS, but it was inferior to DES (7). However, the risk of heterogeneity is high compared with DCB+BMS and DES strategies, and no consistent conclusion was reached in the included trials. In addition, some preliminary studies have shown that DCB only is not inferior to DES in patients with small vessel lesions (8–11). Meanwhile, the efficacy of DCB only was also researched in patients with a high risk of bleeding, acute coronary syndrome, and bifurcation (12–14).

Therefore, stent implantation as the standard strategy for *de novo* coronary artery diseases is challenged by DCB approaches. This network meta-analysis aims to explore the efficacy of DCB approaches (DCB+BMS and DCB only) for *de novo* coronary artery lesions based on network comparison under the framework of the Bayesian network.

## Method

### Data Source

This Bayesian network meta-analysis was implemented by the Preferred Reporting Items for Systematic Reviews and Meta-Analysis (PRISMA) and the PRISMA extension statement for network meta-analysis (15, 16). PubMed, EMBASE, and Cochrane Library databases were retrieved to obtain the relevant trials, including a comparison of DCB approaches and stent implantation strategies from inception to 1 June 2021. In addition, we also screened the abstracts of the scientific conference and related systematic reviews. The major search terms of PubMed were as follows: “*de novo* lesion” or “small coronary artery disease” or “acute myocardial infarction” and “drug-coated balloon” or “drug-eluting balloon” or “paclitaxel-eluting balloon” or “paclitaxel-coated balloon” or “sirolimus coated balloon” or “sirolimus-eluting balloon” and “randomized controlled trials,” with no language restrictions. The details of the search strategy are summarized in Supplementary Tables S1–S3. An update reminder for PubMed was created to keep up with the latest research. This study did not require special ethical permission, as it is a secondary literature study of published randomized controlled trials. The study protocol was registered in PROSPERO (CRD 42021257567).

### The Inclusion and Exclusion Criteria as Well as Outcomes

The inclusion criteria of this study met the following requirements: (a) patients with *de novo* coronary artery diseases; (b) compared the DCB approaches (DCB only or DCB+BMS) and stent implantation (BMS or DES); (c) randomized controlled trials. The exclusion criteria included (a) investigated the efficacy of DCB in the in-stent restenosis; (b) bioabsorbable scaffolds; (c) comparison of different types of stents; (d) reduplicate report and insufficient data from original studies. MACE were defined as the primary outcome, while the clinical outcomes included target lesion revascularization (TLR), all-cause death, and myocardial infarction, and the angiographic outcomes included in-segment LLL and binary restenosis (BR).

### Data Extraction

The two investigators (Peng-Yu Zhong and Ying Ma) initially independently screened the titles and abstracts after the duplicate studies were eliminated by the Endnote software. The full text of the relevant literature was screened out, and the eligible trials were selected according to the inclusion and exclusion criteria. The possible disagreements shall be settled by third-party (Yao-Sheng Shang, Nan Bai, and Ying Niu). In addition, two researchers independently extracted the baseline characteristics and the data of outcomes. The discrepancy was resolved through negotiation with Zhi-Lu Wang.

### Assessment of Quality, GRADE Quality of Evidence, and Publication Bias

The risk of bias in the included randomized controlled trials was evaluated by two researchers according to the *Cochrane Handbook for Systematic Reviewers*, randomized controlled trials risk of the bias assessment tool. Grades of Recommendations Assessment, Development and Evaluation (GRADE) was applied to evaluate the quality of each outcome according to direct, indirect, and network comparison, respectively (17, 18). In addition, the publication bias will be assessed by the visual funnel plot.

### Statistical Analysis

The R version 4.0.1 and JAGS-4.2.0 software were used for statistical analysis in this network meta-analysis. Markov chain Monte Carlo methods and GeMTC package (version 0.8-8) were applied in the R software. First, the convergence of the network model was achieved by 10,000 iterations, of which the degree can be assessed by convergence and trace plot. Then, 50,000 iterations (four Markov chains in total) were run to estimate the parameters. In addition, the odds ratio (OR) and 95% confidence intervals (95% CIs) were calculated using the fixed-effect model, which excluded one that was regarded as statistically significant. For a continuous variable, mean differences (MDs) with standard deviations were presented as summary statistics. The heterogeneity was assessed by the chi-square test; *I*^2^ is applied to judge the degree of heterogeneity, in which <25%, 25%−50%, and >50% represents low, moderate, and high degrees of heterogeneity, respectively (19). Finally, the consistency between direct and indirect sources of evidence was measured by the node-splitting method. The ranking probability plot was used to assess the impact of different strategies for outcome events (20). The Stata 14.1 software was applied to draw reticular relationship plots and funnel plots for each intervention.

The subgroup analysis of patients with acute coronary syndrome was conducted by the published data of the included trials to adequately consider the influence of different presentations. In addition, a series of sensitivity analysis were performed. For example, BMS, first-generation DES, and first-generation DCB were rarely applied in clinical practice; we conducted the sensitivity analysis to explore the robustness of the findings by excluding related trials. The network meta-regression was performed to explore the potential impact of different *de novo* lesions.

## Results

### Search Results and Study Characteristics

**Figure 1:**
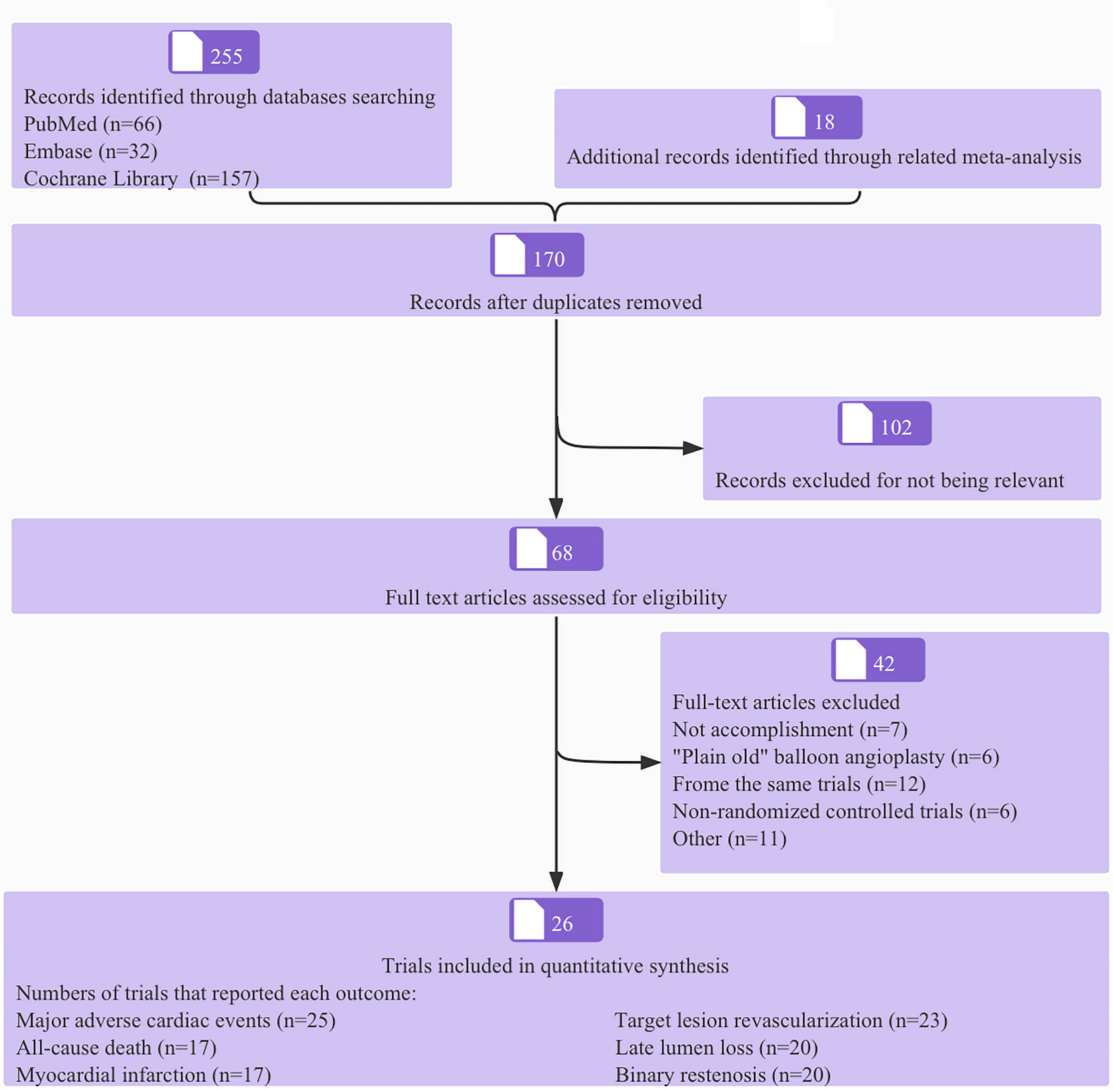
Flow diagram of literature search.

**Figure 2:**
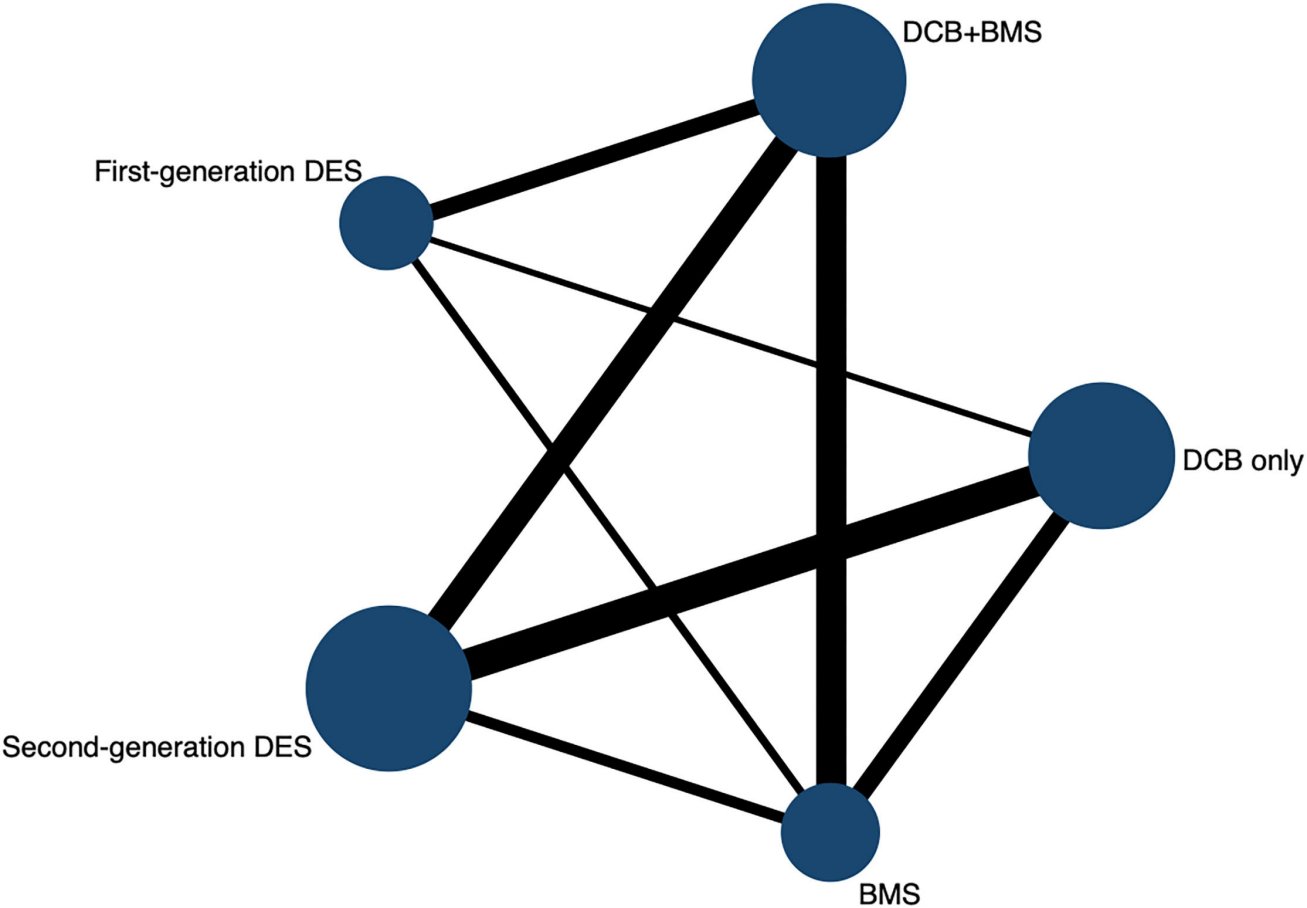
Network evidence plot.

The process of literature screening and trial selection is shown in Figure 1. Of 255 articles, 170 were screened after duplicates were removed, 102 articles were excluded at the title and abstract level, and an additional 68 full-text articles were removed based on selection criteria. Notably, twenty-six trials encompassing 4,664 patients were ultimately included in the network meta-analysis; a reticular relationship plot is shown in Figure 2. All the related references of the included trials are shown in the Supplementary Material. The characteristics of included trials are summarized in Table 1. DCB-only approach included 12 trials and 1,176 patients, and the DCB+BMS group included 14 trials and 1,066 patients. Of note, ten trials with 536 patients referred to BMS strategy, the first-generation DES strategy was applied in ten trials and 813 patients, and the second-generation DES strategy was used for 11 trials and 1,073 patients. The majority of trials were open-label, non-inferiority trials.

**Table 1:** Characteristics of included trials.

**Trials**	**Indication**	**DCB**	**Strategies**					**Clinical follow up (months)**	**Angiographic follow up (months)**
			**DCB only**	**DCB+BMS**	**1st DES**	**2nd DES**	**BMS**		
PICCOLETO	Small-vessel disease	Dior	29		31			9	6
BELLO	Small-vessel disease	IN. PACT Falcon	90		92			36	6
Nishiyama et al.	*de novo* lesions	SeQuent Please	30			30		8	8
Gobic et al.	Myocardial infarction	SeQuent Please	41			37		6	6
BASKET-SMALL-2	Small-vessel disease	SeQuent Please	382			376		12	NR
PICCOLETO II	Small-vessel disease	Elutax SV/Emperor	118			114		6	6
RESTORE SVD	Small-vessel disease	Restore DCB	116			114		12	9
REVELATION	Myocardial infarction	Pantera Lux	60			60		9	9
Shin et al.	High bleeding risk	SeQuent Please	20				20	12	9
DEBUT	High bleeding risk	SeQuent Please	102				106	9	NR
PEPCAD NSTEMI	Myocardial infarction	SeQuent Please	104			51	60	9	NR
Yu et al.	*de novo* lesions	SeQuent Please	84			79			
Ali et al.	*de novo* lesions	SeQuent Please		45	39			9	9
DEBIUT	Bifurcation	DOIR-I		40	37		40	18	6
DEB-AMI	STEMI	DOIR-II		50	49		51	6	6
Besic et al.	NSTE-ACS	Elutax/SeQuent Please		41			44	6	6
IN-PACT CORO	*de novo* lesions	IN-PACT Falcon		20			10	12	6
PEPCAD III	*de novo* lesions	NR		312	325			9	9
Liistro et al.	*de novo* lesions	Elutax		59		66		9	9
BABILON	*de novo* lesions	SeQuent Please		52		56		24	9
Poerner et al.	*de novo* lesions	SeQuent Please		51	48			6	6
Clever et al.	*de novo* lesions	NR		27	25		25	9	9
PEBSI	STEMI	SeQuent Please		110			112	12	9
DEB first	de novo lesions	SeQuent Please		90		90		12	9
Zurakowsk et al.	*de novo* lesions	SeQuent Please		102	100			9	9
Herdeg et al.	*de novo* lesions	GENIE Acrostak		67	67		68	6	6

The baseline characteristics of patients are shown in Supplementary Table S4. The average age was 77 years in the DEBUT trial, but it ranged from 55 to 68 years in the other trials. The proportion of men ranged from 63% to 86.7%. The proportion of patients with diabetes was the highest in the study by Ali et al. (100%), and the lowest in the DEB-AMI trial (7.3%). Patients in the trial by Poerner et al. and Besic et al. were accompanied by hypertension, but patients with hypertension accounted for 30.8–90.9% in the other trials. All subjects were patients with acute coronary syndrome in six trials; the proportion of patients with acute coronary syndrome increased from 23.1 to 89% in the other trials. The proportion of patients with multi-vessel disease was 30–63.2%.

### Risk of Bias and Publication Bias

The results of risk of bias and publication bias are shown in Supplementary Table S5 and Supplementary Figure S1, respectively. The majority of included trials had a low risk of bias in sequence generation and allocation concealment (69 and 65%, respectively). A half of the included trials had a high risk of bias for blinding and others had a low risk of bias for blinding. Of note, 85% trials had a low risk of detection bias, and 69% trials had a low risk of attrition bias. The publication bias evaluation showed that the spots of the funnel plot were symmetrically distributed in each outcome. Therefore, no publication bias was found in this study.

### Clinical Outcomes

**Figure 3:**
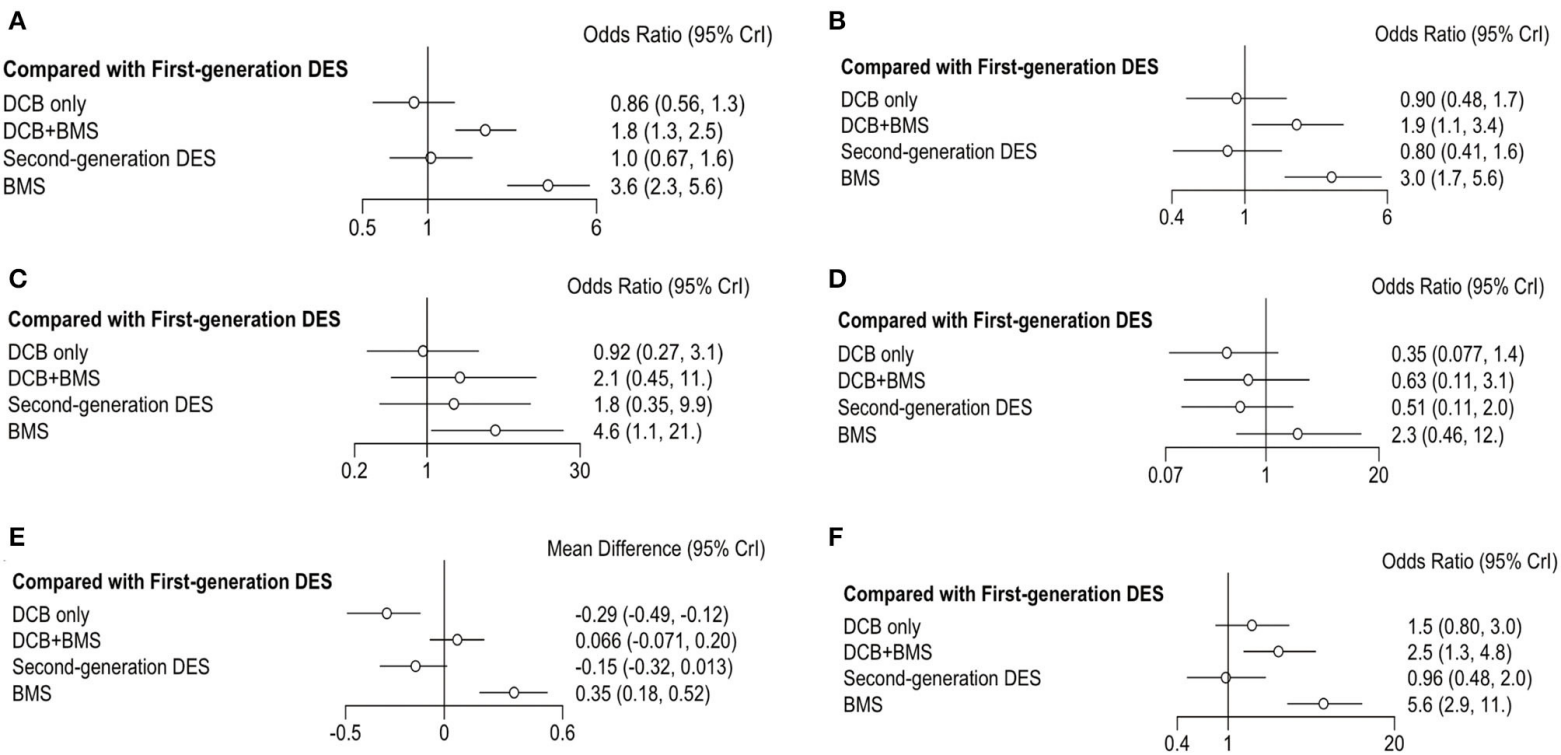
Forest plots of entire cohort compared with first-generation DES. **(A)** MACE; **(B)** TLR; **(C)** all-cause death; **(D)** myocardial infraction; **(E)** LLL; **(F)** BR.

**Figure 4:**
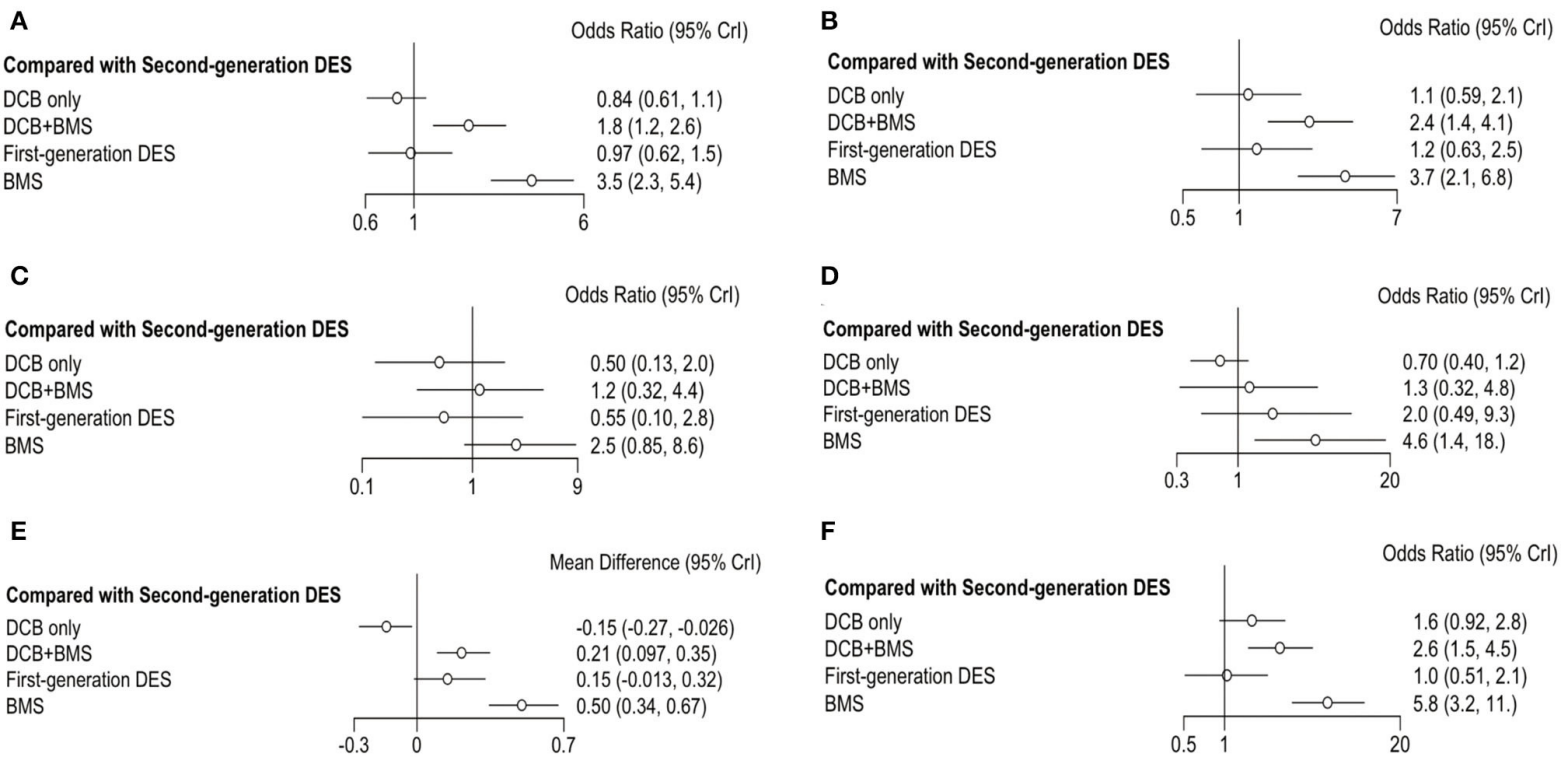
Forest plots of entire cohort compared with second-generation DES. **(A)** MACE; **(B)** TLR; **(C)** all-cause death; **(D)** myocardial infraction; **(E)** LLL; **(F)** BR.

The convergence and trace plots of each outcome are shown in Supplementary Figures S2, S3, which suggested that there was a good degree of convergence. The forest plot shows the OR and 95% CI of each strategy compared with first-generation (Figures 3A–D) and second-generation DES (Figures 4A–D). The data of comparison between any two strategies are summarized in Supplementary Table S6. In addition, the assessment of heterogeneity is also summarized in Supplementary Table S7 and no significant heterogeneity is found (*P*_heterogeneity_ > 0.05).

The primary outcome was reported in 25 trials, which consisted of all-cause death, myocardial infarction, and TLR. DCB only had a similar incidence of MACE than first-generation DES (OR 0.86, 0.56–1.33, *I*^2^ = 86%, *P*_heterogeneity_ = 0.76) and second-generation DES (OR 0.84, 0.61–1.1, *I*^2^ = 0%, *P*_heterogeneity_ = 0.37). However, DCB+BMS increased the risk of MACE compared with first-generation DES (OR 1.8, 1.3–2.5, *I*^2^ = 0%, *P*_heterogeneity_ = 0.75) and second-generation DES (OR 1.8, 1.2–2.6, *I*^2^ = 35%, *P*_heterogeneity_ = 0.76).

TLR was reported in 23 trials; the result showed that the incidence of TLR in DCB+BMS was significantly higher than that in first-generation DES (OR 1.9, 1.1–3.4, *I*^2^ = 2%, *P*_heterogeneity_ = 0.26) and second-generation DES (OR 2.40, 1.40–4.10, *I*^2^ = 37%, *P*_heterogeneity_ = 0.51). However, DCB not only increased this risk of TLR, compared with first-generation DES (OR 0.90, 0.48–1.7, *I*^2^ = 84%, *P*_heterogeneity_ = 0.55) and second-generation DES (OR 1.10, 0.59–2.10, *I*^2^ = 30%, *P*_heterogeneity_ = 0.51), but also it only was significantly superior to DCB+BMS (OR 0.44, 0.22–0.84) and BMS (OR 0.28, 0.14–0.55).

The incidence of all-cause death and myocardial infarction in clinical outcomes was reported in 16 and 17 trials, respectively. Compared with first-generation DES, DCB only (OR 0.92, 0.27–3.1, *I*^2^ = 7%, *P*_heterogeneity_ = 0.87) and DCB+BMS (OR 2.1, 0.45–11, *I*^2^ = 2%, *P*_heterogeneity_ = 0.26) had a similar risk of all-cause death. Similarly, both DCB only (OR 0.5, 0.13–2.0, *I*^2^ = 0%, *P*_heterogeneity_ = 0.97) and DCB+BMS (OR 1.2, 0.32–4.4, *I*^2^ = 0%, *P*_heterogeneity_ = 0.83) did not increase the incidence of all-cause death compared with second-generation DES. In addition, DCB only can significantly reduce the incidence of all-cause death than BMS (OR 0.20, 0.07–0.52), but there was no statistical difference between DCB only and DCB+BMS (OR 0.43, 0.10–1.80).

DCB only (OR 0.35, 0.08–1.36, *I*^2^ = 66%, *P*_heterogeneity_ = 0.88) and DCB+BMS (OR 0.63, 0.11–3.12, *I*^2^ = 47%, *P*_heterogeneity_ = 0.93) had a similar incidence of myocardial infarction as first-generation DES. Similarly, both DCB only (OR 0.70, 0.40–1.20, *I*^2^ = 66%, *P*_heterogeneity_ = 0.88) and DCB+BMS (OR 1.30, 0.32–4.80, *I*^2^ = 0%, *P*_heterogeneity_ = 0.69) also had similar risk than second generation. Meanwhile, DCB only was significantly superior to BMS in reducing the risk of myocardial infarction (OR 0.15, 0.04–0.48). However, there are no statistically significant differences between DCB only and DCB+BMS (OR 0.55, 0.14–2.30).

### Angiographic Outcomes

Angiographic outcomes included LLL and BR; the forest plot shows the OR or MD of each strategy comparing first-generation (Figures 3E,F) with second-generation DES (Figures 4E,F). The LLL was reported in 20 trials, and the results showed that DCB-only strategy can significantly reduce the risk of LLL compared with first-generation DES (MD −0.29, −0.49 to −0.12, *I*^2^ = 84%, *P*_heterogeneity_ = 0.55), DCB+BMS (MD −0.36, −0.54 to −0.21), BMS (MD −0.65, −0.84 to−0.47), and second-generation DES (MD −0.15, −0.27 to−0.026, *I*^2^ = 43%, *P*_heterogeneity_ = 0.53). Meanwhile, the LLL in DCB+BMS was similar to the first-generation DES (MD 0.066,−0.071 to 0.2, *I*^2^ = 84%, *P*_heterogeneity_ = 0.55). However, DCB+BMS had a higher risk of LLL than second-generation DES (MD 0.21, 0.097–0.35, *I*^2^ = 83%, *P*_heterogeneity_ = 0.69).

The incidence of BR as an angiographic outcome was reported in 20 trials, which showed that DCB only was associated with a similar incidence of BR in first-generation DES (OR 1.5, 0.8–3.0, *I*^2^ = 0% *P*_heterogeneity_ = 0.05) and second-generation DES (OR 1.6, 0.92–2.8, *I*^2^ = 0% *P*_heterogeneity_ = 0.11). Conversely, DCB+BMS had a higher incidence of BR than first-generation DES (OR 2.5, 1.3–4.8, *I*^2^ = 26% *P*_heterogeneity_ = 0.39) and second-generation DES (OR 2.6, 1.5–4.5, *I*^2^ = 0% *P*_heterogeneity_ = 0.13).

### Ranking of Treatment Strategies

**Figure 5:**
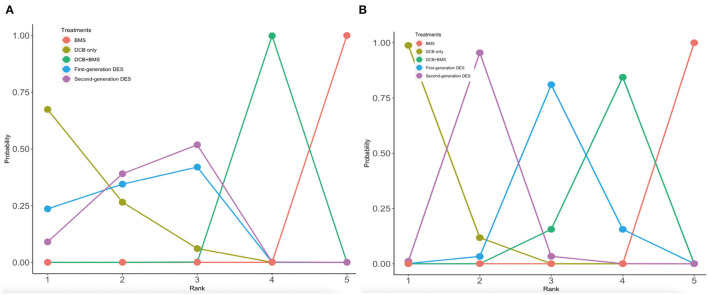
Rankograms on broken line graph from MACE and LLL. **(A)** MACE; **(B)** LLL. The 1–5 on X axial refers to the rank from the best to the worst. The number on y axial refers to the probability of rank.

Rankograms on the histogram and broken line graph are shown in Figure 5 and Supplementary Figure S4. DCB only was ranked the best strategy among MACE (probability of rank, 67%), all-cause death (probability of rank, 46%), myocardial infarction (probability of rank, 69%), and LLL outcomes (probability of rank, 99%). The second-generation DES was ranked the best strategy for both TLR (probability of rank, 54%) and BR outcomes (probability of rank, 54%). In addition, BMS was ranked the worst strategy among all outcomes. In the incidence of primary clinical outcomes, the second-generation DES was similar to the first-generation DES, which was ranked the second strategy. In the LLL outcome, the rank probability of the best strategy was DCB only, the second-generation DES, the first-generation DES, and the DCB+BMS and BMS, respectively.

### Subgroup Analysis, Sensitivity Analysis, and Network Meta-Regression

Patients with acute coronary syndrome could be associated with a higher risk of acute vascular occlusion. In addition, SeQuent Please DCB was the most common type of DCB in the included trials. Therefore, we performed the subgroup analysis of patients with acute coronary syndrome and patients who applied SeQuent Please DCB (Supplementary Table S8). In the subgroup of patients with acute coronary syndrome, a total of six trials were included and four strategies were compared (DCB only, DCB+BMS, DES, and BMS). The results showed that the risk of MACE in acute coronary syndrome patients receiving DCB strategy was similar to that of DES strategy (OR 0.80, 0.31–2.11), but the risk of LLL was higher (MD 0.33, 0.14- 0.51). The subgroup of patients implanted SeQuent Please DCB, and sensitivity analysis does not affect the evaluation of clinical and angiographic outcomes (Supplementary Table S9). We conducted a network meta-regression to explore the impact of different types of *de novo* lesions, which was shown in Supplementary Table S10. All the values of bate included zero, which suggested that the different *de novo* lesions did not influence the robustness of the entire model.

### Network Consistency and GRADE Evidence Quality

The results of the inconsistency test and the assessment of GRADE evidence quality are shown in Supplementary Table S11. The direct evidence comparison and indirect evidence comparison were consistent for the majority of outcomes. That is, the majority of outcomes had a moderate and high evidence quality. However, the outcome of all-cause death was inconsistent when the first-generation DES was compared with DCB only (*P* = 0.01), BMS was compared with DCB only (*P* = 0.01), and DCB+BMS was compared with the first-generation DES (*P* = 0.02). In addition, the inconsistency was found in myocardial infarction when BMS was compared with DCB only (*P* = 0.01) and BMS was compared with the first-generation DES (*P* = 0.01).

## Discussion

The principal results of this Bayesian network meta-analysis suggest that DCB-only strategy was associated with a similar incidence of clinical outcomes compared with the first-generation or second-generation DES. In addition, DCB only is associated with a lower risk of LLL compared with other strategies. The incidence of MACE, TLR, LLL, and BR for DCB+BMS strategy is superior to BMS strategy but inferior to DES. The subgroup analysis suggests that DCB-only strategy was associated with a higher risk of LLL compared with DES strategy. These findings are highly consistent among the direct, indirect, and network comparisons.

Coronary artery diseases have become one of the leading causes of death in the world, and stent implantation is the main therapeutic strategy. In the 1980s, BMS was applied to the coronary artery to resolve the problem of acute vascular occlusion, but 20–30% of restenosis is still caused by neo-intimal hyperplasia (21, 22). In 2003, the first-generation DES was introduced into clinical practice and anti-proliferation agents were transferred to the lesion, which significantly reduced the incidence of restenosis and TLR (23). However, first-generation DES was associated with a higher incidence of late definite stent thrombosis at 12–15 months after implantation compared with BMS (24). Therefore, different from the dual antiplatelet therapy (DAPT) of 1 month in patients with BMS implantation, the DAPT duration of patients following DES was recommended to be 6–12 months (4). Meanwhile, DAPT lasting for 1 year also increases the health economic burden and clinical bleeding risk. In addition, the second-generation DES may not provide an effective therapeutic strategy for small vessels due to the risk of LLL caused by in-stent restenosis (25). Therefore, there are many limitations to the application of DES in patients with a high risk of bleeding. However, with the Department of Stent Technology and implantation technique, new-generation DES can be applied to patients with small vessel lesion and a high risk of bleeding. The BIOFLOW trial showed that bioresorbable polymer DES had similar efficacy and lower TLR compared with durable polymer DES for small vessel lesion (26). In addition, the TICO trial suggested that 1-month dual antiplatelet therapy is effective for patients with acute coronary syndrome after bioresorbable polymer DES was implanted (27). On the contrary, the stent implantation strategy aims to resolve the problem of acute vessel occlusion through the support of a stent, but various factors still need to be solved, such as slow drug release, polymer-induced inflammation, endothelial dysfunction, and coronary vasoconstriction disturbance (28, 29). Therefore, the concept of “leave-nothing-behind strategy” has become a hot spot in the field of intervention. Reducing the implantation of percutaneous coronary intervention can bring more net clinical benefits.

Different from DES, DCB also carried hydrophilic polymer and anti-proliferation agents, but there was no metal platform. Therefore, it directly inhibited the process of neointimal hyperplasia and negative remodeling (30). The study by Wańha et al. (31) showed that paclitaxel DCB has comparable long-term results compared with thin drug-eluting stents for in-stent restenosis. Although the application of DCB-only strategy in patients with in-stent restenosis has been extensively investigated, the application of DCB approaches in *de novo* lesions lacks the evidence of based-evidence medicine. On the one hand, the DCB+BMS strategy retained the metal struts of BMS to prevent acute post-angioplasty recoil and supplemented local release anti-proliferative agents by combining with DCB. The study by Herdeg et al. first explored the efficacy of the DCB+BMS approach; the result showed that the incidences of myocardial infarction (*P* = 0.13), death (*P* = 0.33), and TLR (*P* = 0.2) in DCB+BMS strategy was similar to those in BMS and the first-generation DES strategies (32). On the other hand, the supporting role of the stent is temporary because vascular reconstitution may be completed in the first 6–9 months after DES implantation (33). Interventional therapy remains challenging for small-vessel disease (vessels <2.75 or <3.0 mm), but the application of DCB has been confirmed in a series of randomized controlled trials (8–10). The results of the BASKET-SMALL 2 study demonstrated that DCB strategy was not inferior to the second-generation DES in the small-vessel diseases (11). For bifurcation lesions, DEBIUT by Stella et al. suggested that the angiographic outcome of the DES approach was significantly superior to that of DCB+BMS and BMS approaches (14). In addition, DCB-only strategy was also investigated in patients with acute coronary syndrome. All the related trials demonstrated that DCB only was not inferior to DES strategies, which was also consistent with the subgroup analysis of this study (13).

This study is the first network meta-analysis to explore the efficacy of two DCB approaches for coronary artery *de novo* lesion based on the Bayesian model. A meta-analysis by Cui et al. compared DCB+BMS with stent implantation strategies; the results suggested that DCB+BMS strategy was poorer than DES alone in outcomes of LLL (MD 0.20, 0.07–0.33, *P* = 0.003) and MACE (OR 1.94, 1.24–3.05, *P* = 0.004) (7). However, DCB+BMS strategy can significantly reduce the incidence of MACE (OR, 0.67, 0.45–0.99, *P* = 0.04). Another meta-analysis compared DCB only with stents strategies for *de novo* coronary artery lesions, which showed that DCB only was associated with similar clinical outcomes and lower risk of LLL (MD, −0.17, −0.24 to −0.1, *P* < 0.0001) compared with control group (34). Similarly, the rankogram of this study shows that DCB only is the best strategy to reduce the risk of LLL.

This network meta-analysis favors that DCB only is used to reduce the risk of LLL and has comparable clinical outcomes compared with DES strategies. However, the subgroup analysis of patients with acute coronary syndrome shows that DCB strategy only is associated with a higher risk of LLL than DES strategy. This inconsistency may be due to the high risk of acute vessel occlusion in patients with acute coronary syndrome. In addition, this result is consistent with the DCB consensus in 2020, which suggests that DCB-only strategy should be considered except for the patients with a high risk of acute vessel occlusion or unfavorable long-term results (35). Therefore, optimal lesion preparation is crucial to the outcome of DCB interventional therapy. The result of optimal balloon angioplasty should be confirmed before DCB delivery. The DCB interventional therapy was recommended under acceptable angiographic results, including no flow-limiting dissections residual stenosis ≤ 30% and FFR >80% (35). Moreover, shortening delivery time and sufficient inflation time were also essential for ensuring the efficacy of DCB (35).

DCB+BMS strategy was significantly superior to BMS alone, but it was inferior to DES strategy in the majority of trials. Although the second-generation DES was widely applied to most patients undergoing percutaneous coronary intervention (36), BMS was still used for those patients with a high risk of bleeding. Therefore, both DCB+BMS and DCB only can be the ideal strategy for those patients aiming to minimize the duration of antiplatelet therapy and improve safety. For patients with a high risk of bleeding, the DEBUT trial demonstrated the efficacy of DCB-only strategy (12). However, there is no related trial to testify the efficacy of the DCB+BMS strategy in patients with a high risk of bleeding. Similarly, the LEADERS FREE trial was the first randomized controlled trial to testify the efficacy and safety of 1-month DAPT after polymer-free, biolimus A9-eluting drug-coated stent implanted in patients with a high risk of bleeding (37). Therefore, the optimal strategy of relevant evidence-based medical evidence for patients with a high risk of bleeding needs to be further explored.

DCB+BMS strategy was associated with more pronounced neointimal proliferation compared with DES (38), which may be related to the interaction between BMS and DCB strategies. According to a series of previous studies, the sequence of DCB and BMS is an important factor. Although DCB used before BMS implantation has been widely applied in clinical practice, the stent may be partially implanted outside the DCB-treated segment and increase the risk of geographical mismatch. In contrast, DCB applied after BMS implantation may affect the drug delivery due to the interposition of the stent struts (39). Therefore, under the situation of complete drug release, the matching of the DCB-treated segment with the position of BMS implantation may be essential to ensure the effectiveness of this strategy.

## Limitations

This network meta-analysis has several limitations. First, the Bayesian network meta-analysis is based on study-level data and the majority of trials are open-label, which may result in the risk of bias. Second, the direct comparison between DCB only and DCB+BMS strategies is deficient in the included trials, while the comparison between the two strategies is only based on network frame. Moreover, *de novo* coronary artery lesions included a series of diseases, and there are not enough trails to conduct more subgroup analysis, such as patients with small-vessel diseases and a high risk of bleeding. Finally, although it is not controversial whether patients need to receive DAPT, the optimal duration of DAPT after DCB approaches is uncertain.

## Conclusion

DCB only is associated with similar efficacy and lower risk of LLL compared with DES in the entire group. However, DCB only is associated with a higher risk of LLL than DES in patients with acute coronary syndrome. In addition, the DCB+BMS strategy is superior to BMS alone and inferior to DES, but it may be also a better choice for patients with a high risk of bleeding. Finally, DCB only and DCB+BMS approaches may be a good choice for patients with a high risk of bleeding, which needs to be further studied.

## Data Availability Statement

The original contributions presented in the study are included in the article/Supplementary Material, further inquiries can be directed to the corresponding author.

## Author Contributions

P-YZ: study design, data collection, data analysis, and manuscript. YM: data collection, data analysis, and validation. Y-SS, NB, and YN: data collection and validation. Z-LW: scientific revision of the manuscript. All authors contributed to the article and approved the submitted version.

## Conflict of Interest

The authors declare that the research was conducted in the absence of any commercial or financial relationships that could be construed as a potential conflict of interest.

## Publisher's Note

All claims expressed in this article are solely those of the authors and do not necessarily represent those of their affiliated organizations, or those of the publisher, the editors and the reviewers. Any product that may be evaluated in this article, or claim that may be made by its manufacturer, is not guaranteed or endorsed by the publisher.
