# Supplementary material for: Efficacy of Drug-Coated Balloon Approaches for de novo Coronary Artery Diseases: A Bayesian Network Meta-Analysis
Source: Front Cardiovasc Med. 2022 Jun 21;9:899701. doi: 10.3389/fcvm.2022.899701 (PMC9253576; doi:10.3389/fcvm.2022.899701)
Supplement: Supplementary file 1 [file Data_Sheet_1.doc]

**Supplementary materials**

**Table S1. Search strategy of PubMed database.**

| **#** | **Searches strategy of PubMed** | **Results** |
| --- | --- | --- |
| #1 | (drug coated balloon[Title] OR drug eluting balloon[Title] OR paclitaxel eluting balloon[Title] OR paclitaxel coated balloon[Title] OR sirolimus coated balloon[Title] OR sirolimus eluting balloon[Title] OR DCB[Title] OR PCB[Title] OR SCB[Title]) | 3532 |
| #2 | *de novo* lesion OR *de novo* narrowing OR *de novo* coronary lesion OR *de novo* coronary artery lesion OR *de novo* coronary stenosis OR *de novo* coronary artery disease OR small coronary artery disease OR small coronary vessels OR percutaneous coronary intervention OR Acute Myocardial Infarction | 165824 |
| #3 | "Randomized Controlled Trial"[Publication Type] OR "controll  -ed clinical trial":[tiab] OR "clinical trials, randomized":[tiab] OR "trials, randomized clinical":[tiab] OR "randomized clinical studies":[tiab] | 531119 |
| #4 | "Systematic Review"[Publication Type] OR "Meta-Analysis"  [Publication Type] OR "Review"[Publication Type] OR "meta-  analysis":[ti] OR "systematic review":[ti] OR "literature review"  :[ti] OR "expert consensus":[ti] OR "case report":[ti] | 3168709 |
| #5 | (#1 AND #2 AND #3) NOT #4 | 66 |

**Table S2. Search strategy of EMBASE database.**

| **#** | **Searches strategy of EMBASE** | **Results** |
| --- | --- | --- |
| **#1** | 'drug coated balloon':ti OR 'drug eluting balloon':ti OR 'paclitaxel eluting balloon':ti OR 'paclitaxel coated balloon':ti OR 'sirolimus coated balloon':ti OR 'sirolimus eluting balloon':ti OR dcb:ti OR pcb:ti OR scb:ti | **5184** |
| **#2** | 'de novo lesion':ab,ti OR 'de novo narrowing':ab,ti OR 'de novo coronary lesion':ab,ti OR 'de novo coronary artery lesion':ab,ti OR 'de novo coronary stenosis':ab,ti OR 'de novo coronary artery disease':ab,ti OR 'small coronary artery disease':ab,ti OR 'small coronary vessels':ab,ti OR 'percutaneous coronary intervention':ab,ti OR 'acute myocardial infarction':ab,ti | **99820** |
| **#3** | 'randomized controlled trial'/exp OR 'controlled clinical trial':ab,ti OR 'clinical trials, randomized':ab,ti OR 'trials, randomized clinical':ab,ti OR 'randomized clinical studies':  ab,ti | **665272** |
| **#4** | **#1 AND #2 AND #3** | **32** |

**Table S3. Search strategy of Cochrane Library database.**

| **#** | **Searches strategy of Cochrane Library** | **Results** |
| --- | --- | --- |
| **#1** | (drug coated balloon OR drug eluting balloon OR paclitaxel eluting balloon OR paclitaxel coated balloon OR sirolimus coated balloon OR sirolimus eluting balloon OR dcb OR pcb OR scb):ti | **822** |
| **#2** | (de novo lesion OR de novo narrowing OR de novo coronary lesion OR de novo coronary artery lesion OR de novo coronary stenosisi OR de novo coronary artery disease OR small coronary artery disease OR small coronary vessels OR percutaneous coronary intervention OR acute myocardial infarction):ti,ab,kw | **24491** |
| **#3** | (controlled clinical trial):ti,ab OR (clinical trials, randomized):ti,ab OR (trials, randomized clinical):ti,ab OR (randomized clinical studies):ti,ab OR (randomized):ti OR (randomized controlled trial*):ti,ab OR (randomized clinical trial*):ti,ab | **566056** |
| **#4** | **#1 AND #2 AND #3** | **157** |

**Table S4. Characteristics of Included patients.**

| Trials | Year | Main Country | Age, n (%) | Men, n (%) | DM, n (%) | HTN, n (%) | ACS, n (%) | DLM, n (%) | MD, n (%) |
| --- | --- | --- | --- | --- | --- | --- | --- | --- | --- |
| Herdeg et al. | 2009 | Germany | 65 | 157(77.7) | 72(35.6) | 176(87.1) | - | 146(72.2) | 96(47.5) |
| PICCOLETO | 2010 | Italy | 68 | 44(77.1) | 24(42.1) | 41(71.9) | 31(54.4) | 30(52.6) | 36(63.2) |
| Ali et al. | 2011 | Malaysia | 61 | 64(76.2) | 84(100) | 71(84.5) | - | 63(75) | - |
| DEBIUT | 2012 | Netherlands | 64 | 85(72.6) | 13(11.1) | 67(57.2) | - | 65(55.6) | - |
| DEB-AMI | 2012 | Italy | 59 | 124(82.7) | 11(7.3) | 50(33.3) | 150(100) | 40(26.7) | - |
| PEPCAD III | 2012 | Germany | 66 | 478(75.1) | 174(27.4) | 493(77.5) | - | 424(66.6) | - |
| Liistro et al. | 2013 | Italy | 66 | 107(85.6) | 17(13.6) | 50(40) | 23(18.4) | 16(12.8) | - |
| Clever et al. | 2013 | Germany | 66 | 57(74.0) | 19(24.7) | 70(90.9) | - | 59(76.6) | 28(36.4) |
| BELLO | 2014 | Italy | 66 | 143(78.6) | 74(40.7) | 147(80.8) | 42(23.1) | 144(79.1) | 112(61.5) |
| Besic et al. | 2014 | Croatia | 66 | 68(80) | 25(29.4) | 85(100) | 85(100) | 75(88.2) | - |
| BABILON | 2014 | Spain | 65 | 70(64.8) | 34(31.5) | 67(62.0) | 47(43.5) | 69(63.9) | - |
| Poerner et al. | 2014 | Germany | 69 | 72(72.7) | 47(47.5) | 99(100) | - | 73(73.7) | - |
| IN-PACT CORO | 2015 | Italy | 67 | 26(86.7) | - | 23(76.7) | 0/0 | 24(80) | 9(30) |
| Zurakowski et al. | 2015 | American | 64 | 138(68.3) | 45(22.2) | 169(83.7) | 94(46.5) | 108(53.5) | - |
| Nishiyama et al. | 2016 | Japan | 68 | 44(73.3) | 25(41.7) | 40(66.7) | 60(100) | 47(78.3) | - |
| PEBSI | 2016 | Spain | 61 | 190(85.6) | 37(16.7) | 97(43.7) | - | 102(45.9) | - |
| Gobic et al. | 2017 | Croatia | 55 | 56(71.8) | 6(7.7) | 26(33.3) | 78(100) | 11(14.1) | - |
| DEB first | 2017 | Korea | 62 | 132(73.3) | 54(30) | 65(36.1) | 95(52.8) | 33(18.3) | 100(55.6) |
| BASKET-SMALL-2 | 2019 | Germany | 67 | 557(73.5) | 252(33.2) | 656(86.5) | 214(28.2) | 521(68.7) | - |
| PICCOLETO II | 2019 | Italy | 64/62 | 70/77 | 38/35 | 65/67 | 45/44 | - |  |
| RESTORE SVD | 2019 | China | 60 | 165(71.7) | 94(40.9) | 164(71.3) | 161(70) | 116(50.4) | 93(40.4) |
| REVELATION | 2019 | Netherlands | 57 | 104(86.7) | 12(10) | 37(30.8) | 120(100) | 18(15) | - |
| Shin et al. | 2019 | Korea | 59 | 29(72.5) | 12(30) | 17(42.5) | 14(35) | 20(50) | - |
| DEBUT | 2019 | Finland | 77 | 131(63.0) | 79(38.0) | 185(88.9) | 96(46.2) | 169(81.3) | - |
| PEPCAD NSTEMI | 2019 | Germany | 66 | 141(67.1) | 66(31.4) | 175(83.3) | 210(100) | 100(47.6) | - |
| Yu et al. | 2020 | China | 63 | 118(72.4) | 39(23.9) | 104(63.8) | 145(89.0) | 91(55.8) | 77(47.2) |

Abbreviations: DM, diabetes mellitus; HTN, hypertension; ACS, acute coronary syndrome; DLM, dyslipidaemia; MD, multivessel disease.

**Table S5. The assessment for risk of bias.**

| Trials | Sequence generation | Allocation  concealment | Blinding | Detection  bias | Attrition  bias |
| --- | --- | --- | --- | --- | --- |
| Herdeg et al. | Low | Low | Low | Low | Unclear |
| PICCOLETO | Low | Low | Low | Low | Unclear |
| Ali et al. | Unclear | Unclear | Low | Low | Low |
| DEBIUT | Low | Low | Low | Low | Unclear |
| DEB-AMI | Low | Low | Low | High | Low |
| PEPCAD III | Unclear | Unclear | Low | Low | Low |
| Liistro et al. | Low | Low | High | High | Unclear |
| Clever et al. | Unclear | Unclear | Low | Low | Unclear |
| BELLO | Low | Low | Low | Low | Low |
| Besic et al. | Unclear | Unclear | Low | Low | Unclear |
| BABILON | Low | Unclear | Low | Low | Low |
| Poerner et al. | Unclear | Unclear | Low | High | Low |
| IN-PACT CORO | Unclear | Unclear | High | Low | Low |
| Zurakowski et al. | Unclear | Low | Low | Low | Unclear |
| Nishiyama et al. | Low | Low | High | Low | Low |
| PEBSI | Unclear | Unclear | Low | Unclear | Unclear |
| Gobic et al. | Low | Low | High | Low | Low |
| DEB first | Low | Unclear | High | Low | Low |
| BASKET-SMALL-2 | Low | Low | High | Low | Low |
| PICCOLETO II | Low | Low | High | Low | Low |
| RESTORE SVD | Low | Low | High | Low | Low |
| REVELATION | Low | Low | High | Low | Low |
| Shin et al. | Low | Low | High | Low | Low |
| DEBUT | Low | Low | High | Low | Low |
| PEPCAD NSTEMI | Low | Low | High | Low | Low |
| Yu et al. | Low | Low | High | Low | Low |

**Table S6. The data of network meta-analysis comparing between any two strategies in entire cohort.**

| **MACE** |  |  |  |  |
| --- | --- | --- | --- | --- |
| DCB only | 2.13 (1.42, 3.22) | 1.16 (0.75, 1.78) | 1.19 (0.87, 1.63) | 4.12 (2.66, 6.46) |
| 0.47 (0.31, 0.7) | DCB+BMS | 0.54 (0.39, 0.75) | 0.56 (0.38, 0.82) | 1.94 (1.36, 2.78) |
| 0.86 (0.56, 1.33) | 1.84 (1.34, 2.54) | First-generation DES | 1.03 (0.67, 1.6) | 3.57 (2.32, 5.63) |
| 0.84 (0.61, 1.14) | 1.78 (1.22, 2.61) | 0.97 (0.62, 1.5) | Second-generation DES | 3.45 (2.25, 5.31) |
| 0.24 (0.15, 0.38) | 0.52 (0.36, 0.74) | 0.28 (0.18, 0.43) | 0.29 (0.19, 0.44) | BMS |
| **TLR** |  |  |  |  |
| DCB only | 2.30 (1.20, 4.50) | 1.16 (0.62, 2.19) | 1.00 (0.53, 1.85) | 3.53 (1.83, 7.10) |
| 0.44 (0.22, 0.84) | DCB+BMS | 0.50 (0.28, 0.88) | 0.43 (0.25, 0.72) | 1.54 (1.02, 2.32) |
| 0.9 (0.48, 1.71) | 1.91 (1.14, 3.43) | First-generation DES | 0.84 (0.41, 1.61) | 3.06 (1.70, 5.62) |
| 1.00 (0.54, 1.88) | 2.31 (1.40, 3.92) | 1.17 (0.60, 2.27) | Second-generation DES | 3.57 (2.02, 6.40) |
| 0.28 (0.14, 0.55) | 0.65 (0.43, 0.98) | 0.33 (0.18, 0.59) | 0.28 (0.16, 0.49) | BMS |
| **All-cause death** |  |  |  |  |
| DCB only | 2.32 (0.55, 9.66) | 1.10 (0.32, 3.77) | 2.03 (0.52, 7.56) | 4.96 (1.92, 15.03) |
| 0.43 (0.10, 1.80) | DCB+BMS | 0.47 (0.09, 2.27) | 0.87 (0.23, 3.23) | 2.16 (0.65, 7.74) |
| 0.92 (0.27, 3.11) | 2.11 (0.45, 10.67) | First-generation DES | 1.84 (0.35, 9.92) | 4.56 (1.11, 21.07) |
| 0.49 (0.13, 1.93) | 1.15 (0.31, 4.30) | 0.54 (0.10, 2.87) | Second-generation DES | 2.48 (0.82, 8.35) |
| 0.20 (0.07, 0.52) | 0.46 (0.13, 1.54) | 0.22 (0.05, 0.90) | 0.40 (0.12, 1.22) | BMS |
| **Myocardial infarction** |  |  |  |  |
| DCB only | 1.81 (0.44, 7.09) | 2.83 (0.73, 12.63) | 1.42 (0.82, 2.47) | 6.64 (2.07, 26.16) |
| 0.55 (0.14, 2.30) | DCB+BMS | 1.58 (0.32, 8.49) | 0.78 (0.21, 3.16) | 3.68 (0.92, 17.86) |
| 0.35 (0.08, 1.36) | 0.63 (0.11, 3.12) | First-generation DES | 0.51 (0.11, 2.01) | 2.33 (0.46, 12.42) |
| 0.71 (0.41, 1.22) | 1.27 (0.32, 4.81) | 1.99 (0.49, 9.23) | Second-generation DES | 4.71 (1.43, 18.37) |
| 0.15 (0.04, 0.48) | 0.27 (0.06, 1.09) | 0.43 (0.08, 2.11) | 0.21 (0.05, 0.70) | BMS |
| **LLL** |  |  |  |  |
| DCB only | 0.36 (0.21, 0.54) | 0.29 (0.12, 0.49) | 0.15 (0.03, 0.27) | 0.65 (0.47, 0.84) |
| -0.36 (-0.54, -0.21) | DCB+BMS | -0.07 (-0.2, 0.07) | -0.21 (-0.35, -0.1) | 0.29 (0.14, 0.42) |
| -0.29 (-0.49, -0.12) | 0.07 (-0.07, 0.2) | First-generation DES | -0.15 (-0.32, 0.01) | 0.35 (0.18, 0.52) |
| -0.15 (-0.27, -0.026) | 0.21 (0.1, 0.35) | 0.15 (-0.01, 0.32) | Second-generation DES | 0.5 (0.34, 0.67) |
| -0.65 (-0.84, -0.47) | -0.29 (-0.42, -0.14) | -0.35 (-0.52, -0.18) | -0.5 (-0.67, -0.34) | BMS |
| **BR** |  |  |  |  |
| DCB only | 1.61 (0.83, 3.18) | 1.65 (0.33, 1.25) | 0.63 (0.36, 1.08) | 3.66 (1.81, 7.44) |
| 0.62 (0.31, 1.20) | DCB+BMS | 0.40 (0.20, 0.76) | 0.39 (0.22, 0.66) | 2.26 (1.50, 3.43) |
| 1.55 (0.80, 3.04) | 2.49 (1.32, 4.88) | First-generation DES | 0.96 (0.48, 1.98) | 5.62 (2.92, 11.24) |
| 1.59 (0.93, 2.78) | 2.58 (1.52, 4.48) | 1.04 (0.51, 2.07) | Second-generation DES | 5.82 (3.23, 10.84) |
| 0.27 (0.13, 0.55) | 0.44 (0.29, 0.67) | 0.18 (0.09, 0.34) | 0.17 (0.09, 0.31) | BMS |

Grey box refers to reference group. Above the leading diagonal are estimates from direct pairwise meta-analyses, below the leading diagonal are estimates from network meta-analyses.

**Table S7. Assessment of heterogeneity for each outcome comparing between entire cohort.**

| Outcomes | Treatments | | *P* | *I*^2^ (%) |
| --- | --- | --- | --- | --- |
| MACE | DCB only | First-generation DES | 0.76 | 86 |
|  | DCB only | Second-generation | 0.37 | 0 |
|  | DCB only | BMS | 0.11 | 37 |
|  | DCB+BMS | First-generation DES | 0.75 | 0 |
|  | DCB+BMS | Second-generation | 0.76 | 35 |
|  | DCB+BMS | BMS | 0.49 | 0 |
|  | First-generation DES | BMS | 0.79 | 18 |
|  | Second-generation DES | BMS | 0.23 | 0 |
| TLR | DCB only | First-generation DES | 0.55 | 84 |
|  | DCB only | Second-generation | 0.51 | 30 |
|  | DCB only | BMS | 0.12 | 50 |
|  | DCB+BMS | First-generation DES | 0.26 | 2 |
|  | DCB+BMS | Second-generation | 0.51 | 37 |
|  | DCB+BMS | BMS | 0.08 | 0 |
|  | First-generation DES | BMS | 0.87 | 7 |
|  | Second-generation DES | BMS | 0.24 | 0 |
| All-cause death | DCB only | First-generation DES | 0.77 | 0 |
|  | DCB only | Second-generation | 0.97 | 0 |
|  | DCB only | BMS | 0.80 | 0 |
|  | DCB+BMS | First-generation DES | 0.17 | 0 |
|  | DCB+BMS | Second-generation | 0.83 | 0 |
|  | DCB+BMS | BMS | 0.70 | 0 |
|  | First-generation DES | BMS | 0.37 | 0 |
|  | Second-generation DES | BMS | 0.86 | 0 |
| Myocardial infraction | DCB only | First-generation DES | 0.88 | 66 |
|  | DCB only | Second-generation | 0.77 | 0 |
|  | DCB only | BMS | 0.13 | 0 |
|  | DCB+BMS | First-generation DES | 0.93 | 47 |
|  | DCB+BMS | Second-generation | 0.69 | 0 |
|  | DCB+BMS | BMS | 0.39 | 23 |
|  | First-generation DES | BMS | 0.27 | 0 |
|  | Second-generation DES | BMS | 0.93 | 0 |
| LLL | DCB only | First-generation DES | 0.46 | NA |
|  | DCB only | Second-generation | 0.53 | 43 |
|  | DCB only | BMS | 0.09 | NA |
|  | DCB+BMS | First-generation DES | 0.44 | 24 |
|  | DCB+BMS | Second-generation | 0.69 | 83 |
|  | DCB+BMS | BMS | 0.61 | 49 |
|  | First-generation DES | BMS | 0.19 | 48 |
|  | Second-generation DES | BMS | 0.50 | 0 |
| BR | DCB only | First-generation DES | 0.05 | 0 |
|  | DCB only | Second-generation | 0.11 | 0 |
|  | DCB only | BMS | 0.30 | NA |
|  | DCB+BMS | First-generation DES | 0.39 | 26 |
|  | DCB+BMS | Second-generation | 0.13 | 26 |
|  | DCB+BMS | BMS | NA | 75 |
|  | First-generation DES | BMS | 0.22 | 0 |
|  | Second-generation DES | BMS | 0.57 | NA |

**Table S8. The results of subgroup analysis.**

**(1) Patients with acute coronary syndrome.**

| **MACE** |  |  |  |
| --- | --- | --- | --- |
| DCB only | 3.05 (1.07, 9.22) | 1.25 (0.47, 3.28) | 4.79 (1.93, 13) |
| 0.33 (0.11, 0.93) | DCB+BMS | 0.41 (0.15, 1.01) | 1.57 (0.89, 2.79) |
| 0.8 (0.31, 2.11) | 2.45 (0.99, 6.46) | DES | 3.85 (1.73, 9.38) |
| 0.21 (0.08, 0.52) | 0.64 (0.36, 1.12) | 0.26 (0.11, 0.58) | BMS |
| **TLR** |  |  |  |
| DCB only | 2.88 (0.25, 37.43) | 0.26 (0.02, 2.19) | 3.34 (0.29, 41.02) |
| 0.35 (0.03, 4.06) | DCB+BMS | 0.09 (0.01, 0.47) | 1.15 (0.62, 2.17) |
| 3.87 (0.46, 51.48) | 10.84 (2.14, 111.7) | DES | 12.59 (2.6, 125.3) |
| 0.30 (0.02, 3.46) | 0.87 (0.46, 1.61) | 0.08 (0.01, 0.38) | BMS |
| **All-cause death** |  |  |  |
| DCB only | 0.6 (0.02, 7.32) | 1.81 (0.38, 8.41) | 3.55 (1.01, 14.63) |
| 1.65 (0.14, 56.88) | DCB+BMS | 2.97 (0.25, 97.18) | 5.75 (0.77, 171.3) |
| 0.55 (0.12, 2.62) | 0.34 (0.01, 4.03) | DES | 1.98 (0.57, 8.15) |
| 0.28 (0.07, 0.99) | 0.17 (0.01, 1.3) | 0.51 (0.12, 1.75) | BMS |
| **LLL** |  |  |  |
| DCB only | -0,18 (-0.34, -0.02) | -0.34 (-0.51, -0.14) | 0.19 (0.12, 0.26) |
| 1.8 (0.02, 0.34) | DCB+BMS | -0.15 (-0.31, 0.01) | 0.37 (0.23, 0.51) |
| 0.33(0.14, 0.51) | 0.15 (-0.01, 0.3) | DES | 0.52 (0.35, 0.7) |
| -0.19 (-0.26, -0.12) | -0.37 (-0.51, -0.23) | -0.52 (-0.69, -0.35) | BMS |

**(2) The patients applied SeQuent Please DCB.**

| **MACE** |  |  |  |  |
| --- | --- | --- | --- | --- |
| DCB only | 1.87 (1.01, 3.48) | 1.7 (0.6, 4.81) | 1.27 (0.88, 1.83) | 4.38 (2.43, 8.23) |
| 0.54 (0.29, 0.99) | DCB+BMS | 0.91 (0.39, 2.07) | 0.68 (0.38, 1.18) | 2.36 (1.31, 4.28) |
| 0.59 (0.21, 1.68) | 1.1 (0.48, 2.59) | First-generation DES | 0.74 (0.28, 2.04) | 2.59 (0.94, 7.32) |
| 0.79 (0.55, 1.14) | 1.48 (0.85, 2.6) | 1.34 (0.49, 3.59) | Second-generation DES | 3.48 (1.19, 6.45) |
| 0.23 (0.12, 0.41) | 0.42 (0.23, 0.76) | 0.39 (0.14, 1.06) | 0.29 (0.16, 0.52) | BMS |
| **TLR** |  |  |  |  |
| DCB only | 11.47 (2.45, 96.86) | 11.19 (1.77, 113.8) | 4.87 (1.09, 37.94) | 16.88 (3.73, 140.3) |
| 0.09 (0.01, 0.41) | DCB+BMS | 0.95 (0.36, 2.45) | 0.43 (0.17, 1.02) | 1.46 (0.69, 3.17) |
| 0.09 (0.01, 0.56) | 1.05 (0.41, 2.77) | First-generation DES | 0.44 (0.12, 1.63) | 1.54 (0.46, 5.34) |
| 0.21 (0.03, 0.92) | 2.35 (0.98, 6.01) | 2.25 (0.61, 8.5) | Second-generation DES | 3.46 (1.18, 10.75) |
| 0.06 (0.01, 0.27) | 0.68 (0.32, 1.45) | 0.65 (0.19, 2.18) | 0.29 (0.09, 0.85) | BMS |
| **Myocardial infarction** |  |  |  |  |
| DCB only | 1.46 (0.13, 15.68) | 1.69 (0.02, 128.4) | 1.34 (0.73, 2.48) | 18.62 (2.75, 577.3) |
| 0.68 (0.06, 7.49) | DCB+BMS | 1.14 (0.03, 44) | 0.91 (0.09, 9.15) | 13.46 (0.62, 798.4) |
| 0.59 (0.01, 46.72) | 0.88 (0.02, 37.8) | First-generation DES | 0.79 (0.01, 61.03) | 12.24 (0.1, 2791) |
| 0.75 (0.4, 1.38) | 1.1 (0.11, 10.74) | 1.26 (0.02, 94.04) | Second-generation DES | 13.96 (1.94, 439.7) |
| 0.05 (0, 0.36) | 0.07 (0, 1.61) | 0.08 (0, 9.61) | 0.07 (0, 0.52) | BMS |
| **LLL** |  |  |  |  |
| DCB only | 1.38 (1.27, 1.5) | 1.43 (1.16, 1.75) | 1.21 (1.14, 1.28) | 2.41 (2.02, 2.86) |
| 0.73 (0.67, 0.79) | DCB+BMS | 1.04 (0.86, 1.25) | 0.88 (0.82, 0.93) | 1.75 (1.49, 2.05) |
| 0.7 (0.57, 0.86) | 0.97 (0.8, 1.17) | First-generation DES | 0.85 (0.7, 1.03) | 1.69 (1.31, 2.16) |
| 0.83 (0.78, 0.88) | 1.14 (1.07, 1.21) | 1.18 (0.97, 1.44) | Second-generation DES | 1.99 (1.68, 2.36) |
| 0.42 (0.35, 0.5) | 0.57 (0.49, 0.67) | 0.59 (0.46, 0.76) | 0.5 (0.42, 0.59) | BMS |
| **BR** |  |  |  |  |
| DCB only | 7.25 (1.39, 46.29) | 8 (0.95, 82.86) | 1.05 (0.34, 3.3) | 171.7 (19.66, 2257) |
| 0.14 (0.02, 0.72) | DCB+BMS | 1.09 (0.28, 4.38) | 0.15 (0.03, 0.48) | 22.46 (5.91, 156.1) |
| 0.12 (0.01, 1.06) | 0.92 (0.23, 3.63) | First-generation DES | 0.13 (0.02, 0.83) | 21.47 (2.94, 215.9) |
| 0.95 (0.3, 2.96) | 6.8 (2.08, 31.68) | 7.61 (1.21, 58.22) | Second-generation DES | 162 (25.43, 1713) |
| 0.01 (0, 0.05) | 0.04 (0.01, 0.17) | 0.05 (0, 0.34) | 0.01 (0, 0.04) | BMS |

**Table S9. The results of sensitivity analysis for network meta-analysis.**

***(1) The* sensitivity analysis by e*xcluding the trials of DEB-AMI, Gobic et al, REVELATION and Shin et al (average age less than 60).***

| **MACE** |  |  |  |  |
| --- | --- | --- | --- | --- |
| DCB only | 2.04 (1.34, 3.1) | 1.19 (0.76, 1.84) | 1.19 (0.87, 1.64) | 4.03 (2.53, 6.43) |
| 0.49 (0.32, 0.74) | DCB+BMS | 0.58 (0.42, 0.81) | 0.58 (0.4, 0.85) | 1.97 (1.34, 2.95) |
| 0.84 (0.54, 1.31) | 1.72 (1.24, 2.4) | First-generation DES | 1.01 (0.64, 1.57) | 3.4 (2.14, 5.47) |
| 0.84 (0.61, 1.16) | 1.71 (1.17, 2.52) | 0.99 (0.64, 1.56) | Second-generation DES | 3.39 (2.16, 5.34) |
| 0.25 (0.16, 0.4) | 0.51 (0.34, 0.74) | 0.29 (0.18, 0.47) | 0.29 (0.19, 0.46) | BMS |
| **TLR** |  |  |  |  |
| DCB only | 1.72 (0.86, 3.45) | 1.2 (0.64, 2.33) | 0.79 (0.4, 1.56) | 2.81 (1.36, 5.9) |
| 0.58 (0.29, 1.16) | DCB+BMS | 0.7 (0.37, 1.3) | 0.46 (0.27, 0.76) | 1.63 (1.04, 2.58) |
| 0.83 (0.43, 1.56) | 1.43 (0.77, 2.68) | First-generation DES | 0.66 (0.32, 1.33) | 2.34 (1.19, 4.64) |
| 1.26 (0.64, 2.51) | 2.18 (1.31, 3.71) | 1.53 (0.75, 3.13) | Second-generation DES | 3.56 (1.97, 6.63) |
| 0.36 (0.17, 0.74) | 0.61 (0.39, 0.96) | 0.43 (0.22, 0.84) | 0.28 (0.15, 0.51) | BMS |
| All-cause death |  |  |  |  |
| DCB only | 2.75 (0.63, 12.18) | 1.26 (0.36, 4.64) | 2 (0.53, 7.55) | 4.42 (1.67, 13.6) |
| 0.36 (0.08, 1.59) | DCB+BMS | 0.47 (0.08, 2.45) | 0.73 (0.19, 2.67) | 1.61 (0.44, 6.14) |
| 0.79 (0.22, 2.8) | 2.14 (0.41, 12.41) | First-generation DES | 1.57 (0.28, 9) | 3.53 (0.77, 16.75) |
| 0.5 (0.13, 1.9) | 1.37 (0.38, 5.31) | 0.64 (0.11, 3.58) | Second-generation DES | 2.21 (0.74, 7.63) |
| 0.23 (0.07, 0.6) | 0.62 (0.16, 2.27) | 0.28 (0.06, 1.3) | 0.45 (0.13, 1.35) | BMS |
| **Myocardial infarction** |  |  |  |  |
| DCB only | 0.9 (0.16, 3.99) | 3.29 (0.79, 19.19) | 1.37 (0.78, 2.37) | 9.15 (2.46, 48.2) |
| 1.12 (0.25, 6.13) | DCB+BMS | 3.78 (0.62, 33.57) | 1.53 (0.36, 8.03) | 10.58 (1.87, 85.06) |
| 0.3 (0.05, 1.27) | 0.26 (0.03, 1.62) | First-generation DES | 0.41 (0.07, 1.91) | 2.78 (0.36, 22.73) |
| 0.73 (0.42, 1.27) | 0.65 (0.12, 2.82) | 2.43 (0.52, 14.63) | Second-generation DES | 6.77 (1.78, 35.54) |
| 0.11 (0.02, 0.41) | 0.1 (0.01, 0.54) | 0.36 (0.04, 2.78) | 0.15 (0.03, 0.56) | BMS |
| **LLL** |  |  |  |  |
| DCB only | 1.29 (1.2, 1.39) | 1.23 (1.13, 1.32) | 1.12 (1.06, 1.18) | 1.77 (1.57, 1.98) |
| 0.77 (0.72, 0.83) | DCB+BMS | 0.95 (0.89, 1) | 0.86 (0.82, 0.91) | 1.37 (1.24, 1.51) |
| 0.82 (0.76, 0.88) | 1.06 (1, 1.12) | First-generation DES | 0.91 (0.85, 0.98) | 1.44 (1.29, 1.61) |
| 0.9 (0.85, 0.95) | 1.16 (1.1, 1.22) | 1.1 (1.02, 1.18) | Second-generation DES | 1.58 (1.42, 1.76) |
| 0.57 (0.5, 0.64) | 0.73 (0.66, 0.81) | 0.69 (0.62, 0.78) | 0.63 (0.57, 0.7) | BMS |
| **BR** |  |  |  |  |
| DCB only | 1.35 (0.65, 2.78) | 0.65 (0.32, 1.29) | 0.58 (0.32, 1.03) | 3.89 (1.76, 8.64) |
| 0.74 (0.36, 1.54) | DCB+BMS | 0.49 (0.22, 1.01) | 0.43 (0.24, 0.74) | 2.89 (1.83, 4.64) |
| 1.54 (0.77, 3.13) | 2.06 (0.99, 4.44) | First-generation DES | 0.9 (0.42, 1.92) | 5.96 (2.73, 13.83) |
| 1.71 (0.97, 3.09) | 2.3 (1.34, 4.09) | 1.12 (0.52, 2.37) | Second-generation DES | 6.66 (3.52, 13.06) |
| 0.26 (0.12, 0.57) | 0.35 (0.22, 0.55) | 0.17 (0.07, 0.37) | 0.15 (0.08, 0.28) | BMS |

***(2) The sensitivity analysis by excluding the trial conducted by Ali et al (all the patients with diabetes mellitus).***

| **MACE** |  |  |  |  |
| --- | --- | --- | --- | --- |
| DCB only | 2.16 (1.43, 3.27) | 1.14 (0.75, 1.76) | 1.2 (0.88, 1.65) | 4.17 (2.67, 6.62) |
| 0.46 (0.31, 0.7) | DCB+BMS | 0.53 (0.38, 0.73) | 0.56 (0.38, 0.82) | 1.93 (1.35, 2.78) |
| 0.88 (0.57, 1.34) | 1.89 (1.36, 2.65) | First-generation DES | 1.05 (0.67, 1.63) | 3.65 (2.38, 5.68) |
| 0.83 (0.61, 1.14) | 1.8 (1.23, 2.64) | 0.95 (0.61, 1.48) | Second-generation DES | 3.47 (2.24, 5.44) |
| 0.24 (0.15, 0.37) | 0.52 (0.36, 0.74) | 0.27 (0.18, 0.42) | 0.29 (0.18, 0.45) | BMS |
| **TLR** |  |  |  |  |
| DCB only | 2.34 (1.21, 4.62) | 1.03 (0.55, 1.94) | 0.95 (0.5, 1.8) | 3.53 (1.79, 7.09) |
| 0.43 (0.22, 0.83) | DCB+BMS | 0.44 (0.23, 0.81) | 0.41 (0.24, 0.67) | 1.5 (0.99, 2.28) |
| 0.97 (0.52, 1.83) | 2.26 (1.24, 4.28) | First-generation DES | 0.92 (0.46, 1.88) | 3.4 (1.81, 6.67) |
| 1.06 (0.56, 2.02) | 2.47 (1.48, 4.22) | 1.09 (0.53, 2.2) | Second-generation DES | 3.71 (2.08, 6.78) |
| 0.28 (0.14, 0.56) | 0.66 (0.44, 1.01) | 0.29 (0.15, 0.55) | 0.27 (0.15, 0.48) | BMS |
| **All-cause death** |  |  |  |  |
| DCB only | 1.28 (0.24, 6.36) | 1.59 (0.44, 6.43) | 1.5 (0.35, 5.77) | 4.09 (1.59, 12.04) |
| 0.78 (0.16, 4.12) | DCB+BMS | 1.25 (0.17, 9.71) | 1.16 (0.29, 4.87) | 3.21 (0.86, 14.5) |
| 0.63 (0.16, 2.29) | 0.8 (0.1, 5.77) | First-generation DES | 0.93 (0.13, 5.67) | 2.57 (0.53, 12.67) |
| 0.67 (0.17, 2.84) | 0.86 (0.21, 3.49) | 1.08 (0.18, 7.42) | Second-generation DES | 2.75 (0.91, 9.95) |
| 0.24 (0.08, 0.63) | 0.31 (0.07, 1.16) | 0.39 (0.08, 1.89) | 0.36 (0.1, 1.1) | BMS |
| **Myocardial infarction** |  |  |  |  |
| DCB only | 1.66 (0.36, 7.1) | 3.01 (0.72, 15.6) | 1.41 (0.82, 2.46) | 6.56 (2.07, 24.84) |
| 0.6 (0.14, 2.81) | DCB+BMS | 1.84 (0.27, 14.52) | 0.85 (0.21, 3.82) | 3.93 (0.94, 20.74) |
| 0.33 (0.06, 1.4) | 0.54 (0.07, 3.68) | First-generation DES | 0.47 (0.09, 2.13) | 2.14 (0.38, 12.57) |
| 0.71 (0.41, 1.22) | 1.18 (0.26, 4.81) | 2.14 (0.47, 11.64) | Second-generation DES | 4.66 (1.44, 17.38) |
| 0.15 (0.04, 0.48) | 0.25 (0.05, 1.06) | 0.47 (0.08, 2.62) | 0.21 (0.06, 0.7) | BMS |
| **LLL** |  |  |  |  |
| DCB only | 1.35 (1.27, 1.44) | 1.25 (1.16, 1.34) | 1.16 (1.11, 1.21) | 1.84 (1.67, 2.04) |
| 0.74 (0.7, 0.79) | DCB+BMS | 0.92 (0.87, 0.98) | 0.86 (0.82, 0.9) | 1.36 (1.25, 1.49) |
| 0.8 (0.75, 0.86) | 1.08 (1.02, 1.14) | First-generation DES | 0.93 (0.87, 1) | 1.48 (1.34, 1.62) |
| 0.86 (0.83, 0.9) | 1.17 (1.11, 1.23) | 1.08 (1, 1.15) | Second-generation DES | 1.59 (1.44, 1.75) |
| 0.54 (0.49, 0.6) | 0.73 (0.67, 0.8) | 0.68 (0.62, 0.74) | 0.63 (0.57, 0.69) | BMS |
| **BR** |  |  |  |  |
| DCB only | 1.35 (0.67, 2.75) | 0.65 (0.32, 1.29) | 0.58 (0.33, 1.02) | 3.89 (1.78, 8.56) |
| 0.74 (0.36, 1.5) | DCB+BMS | 0.48 (0.23, 0.99) | 0.43 (0.25, 0.75) | 2.89 (1.82, 4.66) |
| 1.53 (0.78, 3.09) | 2.06 (1.01, 4.4) | First-generation DES | 0.89 (0.42, 1.92) | 5.95 (2.79, 13.5) |
| 1.71 (0.98, 3.07) | 2.31 (1.33, 4.05) | 1.12 (0.52, 2.38) | Second-generation DES | 6.67 (3.53, 12.94) |
| 0.26 (0.12, 0.56) | 0.35 (0.21, 0.55) | 0.17 (0.07, 0.36) | 0.15 (0.08, 0.28) | BMS |

***(3) The sensitivity analysis by excluding first-generation DES and DCB.***

| **MACE** |  |  |  |
| --- | --- | --- | --- |
| DCB only | 2.69 (1.51, 4.89) | 1.15 (0.82, 1.61) | 5.22 (2.94, 9.65) |
| 0.37 (0.20, 0.66) | DCB+BMS | 0.43 (0.25, 0.71) | 1.94 (1.14, 3.35) |
| 0.87 (0.62, 1.22) | 2.35 (1.40, 4.00) | 2^nd^ DES | 4.56 (2.57, 8.34) |
| 0.19 (0.10, 0.34) | 0.52 (0.30, 0.88) | 0.22 (0.12, 0.39) | BMS |
| **TLR** |  |  |  |
| DCB only | 4.50 (1.68, 13.18) | 0.86 (0.39, 1.91) | 7.29 (2.57, 23.47) |
| 0.22 (0.08, 0.60) | DCB+BMS | 0.19 (0.08, 0.40) | 1.63 (0.82, 3.30) |
| 1.17 (0.52, 2.56) | 5.27 (2.47, 12.24) | 2^nd^ DES | 8.56 (3.36, 23.58) |
| 0.14 (0.04, 0.39) | 0.61 (0.30, 1.22) | 0.12 (0.04, 0.30) | BMS |
| **All-cause death** |  |  |  |
| DCB only | 1.02 (0.11, 7.03) | 2.04 (0.51, 7.87) | 3.12 (1.15, 9.30) |
| 0.98 (0.14, 8.82) | DCB+BMS | 1.97 (0.35, 16.08) | 3.08 (0.57, 23.70) |
| 0.49 (0.13, 1.95) | 0.51 (0.06, 2.88) | 2^nd^ DES | 1.53 (0.47, 5.75) |
| 0.32 (0.11, 0.87) | 0.33 (0.04, 1.76) | 0.65 (0.17, 2.12) | BMS |
| **MI** |  |  |  |
| DCB only | 0.92 (0.10, 6.74) | 1.34 (0.76, 2.38) | 18.01 (2.68, 432.1) |
| 1.09 (0.15, 10.02) | DCB+BMS | 1.47 (0.21, 12.5) | 21.06 (1.27, 896.3) |
| 0.74 (0.42, 1.31) | 0.68 (0.08, 4.66) | 2^nd^ DES | 13.47 (1.92, 341.9) |
| 0.06 (0, 0.37) | 0.05 (0, 0.79) | 0.07 (0, 0.52) | BMS |
| **LLL** |  |  |  |
| DCB only | 0.44(0.33, 0.55) | 0.14 (0.10, 0.19) | 0.91 (0.74, 1.10) |
| -0.44 (-0.55, -0.34) | DCB+BMS | -0.30 (-0.40, -0.20) | 0.47 (0.33, 0.61) |
| -0.14 (-0.19, -0.10) | 0.30 (0.20, 0.40) | 2^nd^ DES | 0.77 (0.60, 0.93) |
| -0.91 (-1.01, -0.74) | -0.47 (-0.61, -0.33) | -0.77 (-0.93, -0.60) | BMS |
| **BR** |  |  |  |
| DCB only | 6,14 (2.16, 20.09) | 0.86 (0.47, 1.58) | 32.91 (8.99, 137.5) |
| 0.16 (0.05, 0.46) | DCB+BMS | 0.14 (0.05, 0.33) | 5.34 (2.51, 12.36) |
| 1.17(0.63, 2.13) | 7.07 (3.03, 19.72) | 2^nd^ DES | 38.19 (12.16, 141.7) |
| 0.03 (0.01, 0.11) | 0.19 (0.08, 0.40) | 0.03 (0.01, 0.08) | BMS |

***(4) The sensitivity analysis by excluding BMS, first-generation DES and DCB.***

| **MACE** |  |  |
| --- | --- | --- |
| DCB only | 2.64 (1.32, 5.4) | 1.11 (0.79, 1.57) |
| 0.38 (0.19, 0.76) | DCB+BMS | 0.42 (0.22, 0.77) |
| 0.9 (0.64, 1.26) | 2.37 (1.3, 4.47) | 2^nd^ DES |
| **TLR** |  |  |
| DCB only | 3.31 (1.02, 10.99) | 0.72 (0.3, 1.63) |
| 0.3 (0.09, 0.98) | DCB+BMS | 0.22 (0.09, 0.48) |
| 1.38 (0.61, 3.32) | 4.55 (2.08, 11.25) | 2^nd^ DES |
| **All-cause death** |  |  |
| DCB only | 1.16 (0.05, 21.55) | 2.06 (0.45, 9.45) |
| 0.86 (0.05, 21.31) | DCB+BMS | 1.76 (0.15, 30.19) |
| 0.49 (0.11, 2.2) | 0.57 (0.03, 6.63) | 2^nd^ DES |
| **MI** |  |  |
| DCB only | 0.9 (0.1, 6.51) | 1.32 (0.75, 2.34) |
| 1.11 (0.15, 10.25) | DCB+BMS | 1.47 (0.22, 12.61) |
| 0.76 (0.43, 1.33) | 0.68 (0.08, 4.5) | 2^nd^ DES |
| **LLL** |  |  |
| DCB only | 0.44(0.32, 0.55) | 0.14 (0.09, 0.19) |
| -0.44 (-0.55, -0.32) | DCB+BMS | -0.29 (-0.39, -0.2) |
| -0.14 (-0.19, -0.09) | 0.3 (0.19, 0.4) | 2^nd^ DES |
| **BR** |  |  |
| DCB only | 5.98 (2.09, 19.24) | 0.86 (0.46, 1.56) |
| 0.17 (0.05, 0.48) | DCB+BMS | 0.14 (0.05, 0.33) |
| 1.17(0.64, 2.17) | 6.98 (2.99, 19.13) | 2^nd^ DES |

**Table S10. The results of network meta-regression.**

| **Outcomes** | **Beta(95%CI)** | **Outcomes** | **Beta(95%CI)** |
| --- | --- | --- | --- |
| **MACE** |  | **MI** |  |
| A VS E | -0.946 (-2.538 to 0.746) | A VS E | -2.574 (-9.078 to 1.395) |
| B VS E | 0.0434 (-0.691 to 1.085) | B VS E | 2.955 (-0.871 to 8.920) |
| C VS E | -0.326 (-0.837 to 1.216) | C VS E | 3.541 (-0.843 to 10.783) |
| D VS E | -0.127 (-1.751 to 1.283) | D VS E | 0.125 (-3.614 to 3.884) |
| **TLR** |  | **LLL** |  |
| A VS E | -0.738 (-3.430 to 1.617) | A VS E | -0.208 (-0.648 to 0.223) |
| B VS E | 0.270 (-0.997 to 1.614) | B VS E | 0.097 (-0.160 to 0.351) |
| C VS E | 0.054 (-1.554 to 1.680) | C VS E | 0.091 (-0.195 to 0.373) |
| D VS E | -2.481 (-6.315 to 0.580) | D VS E | -0.138 (-0.650 to 0.352) |
| **All-cause death** |  | **BR** |  |
| A VS E | 2.018 (-1.984 to 8.147) | A VS E | -0.756 (-5.067 to 2.516) |
| B VS E | -0.491 (-4.368 to 2.868) | B VS E | 0.231 (-1.523 to 1.974) |
| C VS E | 0.724 (-4.250 to 6.788) | C VS E | 0.508 (-1.609 to 2.696) |
| D VS E | 1.092 (-2.114 to 5.355) | D VS E | 0.453 (-3.857 to 5.029) |

**Table S11. The results of node split, the assessment of consistency and GRADE quality of evidence.**

| Outcomes | Interventions | Direct comparison | | Indirect comparison | | Network comparison | | *P* |
| --- | --- | --- | --- | --- | --- | --- | --- | --- |
|  |  | OR(95%CI) | Quality of evidence | OR(95%CI) | Quality of evidence | OR(95%CI) | Quality of evidence |  |
| MACE | C vs A | 1.0, 0.37-2.4 | High | 1.3, 0.56-3.4 | High | 1.2, 0.64-2.1 | High | 0.69 |
|  | D vs A | 1.2, 0.68-2.2 | High | 1.8, 0.63-5.4 | High | 1.3, 0.82-2.1 | High | 0.48 |
|  | E vs A | 8.1, 3-26 | Moderate | 3.3, 1.6-6.7 | High | 4.3, 2.5-8 | Moderate | 0.15 |
|  | C vs B | 0.56, 0.3-1.1 | High | 0.46, 0.12-1.5 | High | 0.55, 0.34-0.89 | High | 0.79 |
|  | D vs B | 0.67, 0.36-1.3 | High | 0.52, 0.2-1.4 | High | 0.6, 0.38-1.0 | High | 0.65 |
|  | E vs B | 2, 1.2-3.7 | High | 2.7, 0.88-9.7 | Moderate | 2.0, 1.3-3.3 | High | 0.65 |
|  | E vs C | 3.5, 1.2-11 | Moderate | 4.0, 1.9-9.5 | Moderate | 3.7, 2.1-6.9 | High | 0.83 |
|  | E vs D | 2.1, 0.96-4.8 | High | 4.4, 2.2-9.4 | Moderate | 3.4, 2.0-5.8 | High | 0.16 |
| TLR | C vs A | 0.82, 0.18-3.3 | High | 1.5, 0.32-7.8 | Moderate | 1.1, 0.39-3.0 | High | 0.52 |
|  | D vs A | 0.76, 0.23-2.5 | High | 1.7, 0.35-13 | Moderate | 0.98, 0.40-2.6 | High | 0.40 |
|  | E vs A | 18, 2.2-5.2e+2 | Low | 2.5, 0.75-8.6 | Moderate | 3.8, 1.5-12 | Moderate | 0.12 |
|  | C vs B | 0.53, 0.15-1.8 | High | 0.31, 0.031-2.7 | High | 0.51, 0.2-1.2 | High | 0.65 |
|  | D vs B | 0.55, 0.2-1.7 | High | 0.25, 0.032-1.7 | High | 0.46, 0.21-1.0 | High | 0.46 |
|  | E vs B | 1.6, 0.81-3.9 | High | 13, 1.1-4.5e+2 | Moderate | 1.8, 0.93-4.0 | Moderate | 0.12 |
|  | E vs C | 4.3, 0.85-25 | Moderate | 3.4, 0.90-18 | High | 3.6, 1.4-11 | Moderate | 0.84 |
|  | E vs D | 2.3, 0.58-11 | Moderate | 6.0, 1.9-22 | Moderate | 3.9, 1.7-11 | Moderate | 0.28 |
| All-cause death | C vs A | 2.1, 0.27-16 | Moderate | 2.6e-8, 1.9e-21-0.17 | Low | 0.87, 0.099-5 | High | 0.01 |
|  | D vs A | 2.0, 0.12-36 | Moderate | 1.0, 0.017-45 | Moderate | 1.8, 0.21-14 | Moderate | 0.76 |
|  | E vs A | 3.2, 0.56-21 | Moderate | 5.8e+5, 39-3.1e+14 | Low | 6.5, 1.5-51 | Moderate | 0.01 |
|  | C vs B | 6.5e-6,9e-18-0.29 | Low | 1.5, 0.072-40 | Moderate | 0.36, 0.019-2.9 | High | 0.02 |
|  | D vs B | 0.47, 0.024-5.9 | High | 1.1, 0.039-33 | Moderate | 0.73, 0.092-4.1 | High | 0.67 |
|  | E vs B | 9.8, 1.1-2.4e+2 | Low | 0.34, 0.01-5.2 | High | 2.7, 0.52-18 | Moderate | 0.05 |
|  | E vs C | 2.2e+3, 0.83-8.2e+12 | Low | 5, 0.61-88 | Low | 7.5, 1.1-1.4e+2 | Low | 0.22 |
|  | E vs D | 2.9, 0.33-44 | Moderate | 6.9, 0.2-3.6e+2 | Low | 3.6, 0.77-33 | Moderate | 0.70 |
| Myocardial infarction | C vs A | 2.0, 0.057-32 | Moderate | 4.1, 0.12-2e+2 | Low | 2.5, 0.27-22 | Moderate | 0.73 |
|  | D vs A | 1.4, 0.24-9 | High | 6.2, 0.13-4.8e+2 | Low | 1.7, 0.42-8.5 | High | 0.46 |
|  | E vs A | 6.1e+8, 32-2.3e+31 | Low | 2.5, 0.11-33 | Moderate | 7.4, 1.1-63 | Moderate | 0.01 |
|  | C vs B | 0.72, 0.051-9 | High | 3.6, 0.067-1.7e+2 | Low | 1.3, 0.13-14 | Moderate | 0.44 |
|  | D vs B | 2.9, 0.28-79 | Moderate | 0.075, 0.0015-2.6 | High | 0.9, 0.15-7.6 | High | 0.08 |
|  | E vs B | 2.5, 0.16-69 | Moderate | 31, 0.63-7.7e+3 | Low | 3.9, 0.56-40 | Moderate | 0.29 |
|  | E vs C | 6.7e-14, 2.6e-41-0.46 | Low | 11, 0.83-4e+2 | Low | 2.9, 0.3-39 | Moderate | 0.01 |
|  | E vs D | 4.2, 0.46-74 | Moderate | 2.1, 0.06-35 | Moderate | 4.4, 0.66-31 | Moderate | 0.68 |
| LLL | C vs A | 0.21, -0.11-0.54 | High | 0.35, 0.12-0.61 | High | 0.29, 0.12-0.49 | High | 0.47 |
|  | D vs A | 0.14, -0.00868-0.28 | High | 0.2, -0.11-0.53 | High | 0.15, 0.024-0.28 | High | 0.72 |
|  | E vs A | 1, 0.54-1.5 | High | 0.59, 0.39-0.78 | High | 0.65, 0.47-0.84 | High | 0.1 |
|  | C vs B | -0.091, -0.23-0.059 | High | -0.15, -0.51-0.17 | High | -0.066, -0.2-0.072 | High | 0.72 |
|  | D vs B | -0.21, -0.38- -0.064 | High | 0.27, -0.65-0.069 | High | -0.21, -0.35- -0.096 | High | 0.75 |
|  | E vs B | 0.28, 0.12-0.43 | High | 0.64, 0.13-1.1 | High | 0.29, 0.15-0.42 | High | 0.17 |
|  | E vs C | 0.23, 0.0018-0.46 | High | 0.45, 0.22-0.65 | High | 0.35, 0.18-0.52 | High | 0.17 |
|  | E vs D | 0.42, 0.13-0.71 | High | 0.55, 0.34-0.79 | High | 0.5, 0.34-0.67 | High | 0.44 |
| BR | C vs A | 0.3, 0.06-1.4 | High | 2.6, 0.41-21 | Moderate | 0.7, 0.2-2.6 | High | 0.07 |
|  | D vs A | 0.87, 0.25-3.1 | High | 0.24, 0.03-2.2 | Moderate | 0.62, 0.21-1.9 | High | 0.28 |
|  | E vs A | 3.2e+8, 5.2-1.4e+23 | Low | 3.3, 0.74-15 | High | 4.2, 1.1-19 | Moderate | 0.04 |
|  | C vs B | 0.48, 0.13-1.7 | High | 0.085, 0.0074-0.7 | High | 0.38, 0.12-1.1 | High | 0.15 |
|  | D vs B | 0.25, 0.063- 0.86 | High | 0.92, 0.067-9.4 | High | 0.34, 0.11- 0.91 | High | 0.32 |
|  | E vs B | 2.4, 0.81-7.5 | High | 4.1e+9, 2.9-1.1e+24 | Low | 2.3, 0.9-6.0 | High | 0.05 |
|  | E vs C | 2.9, 0.56-16 | Moderate | 15, 2.3-1.2e+2 | Low | 5.9, 1.8-22 | Moderate | 0.18 |
|  | E vs D | 4.2, 0.4-0.47 | High | 9.2, 2-55 | Moderate | 6.7, 2.1-25 | Moderate | 0.55 |

A: DCB only; B: DCB+BMS; C: First-generation DES; D: Second-generation DES; E: BMS.

**A** **B**


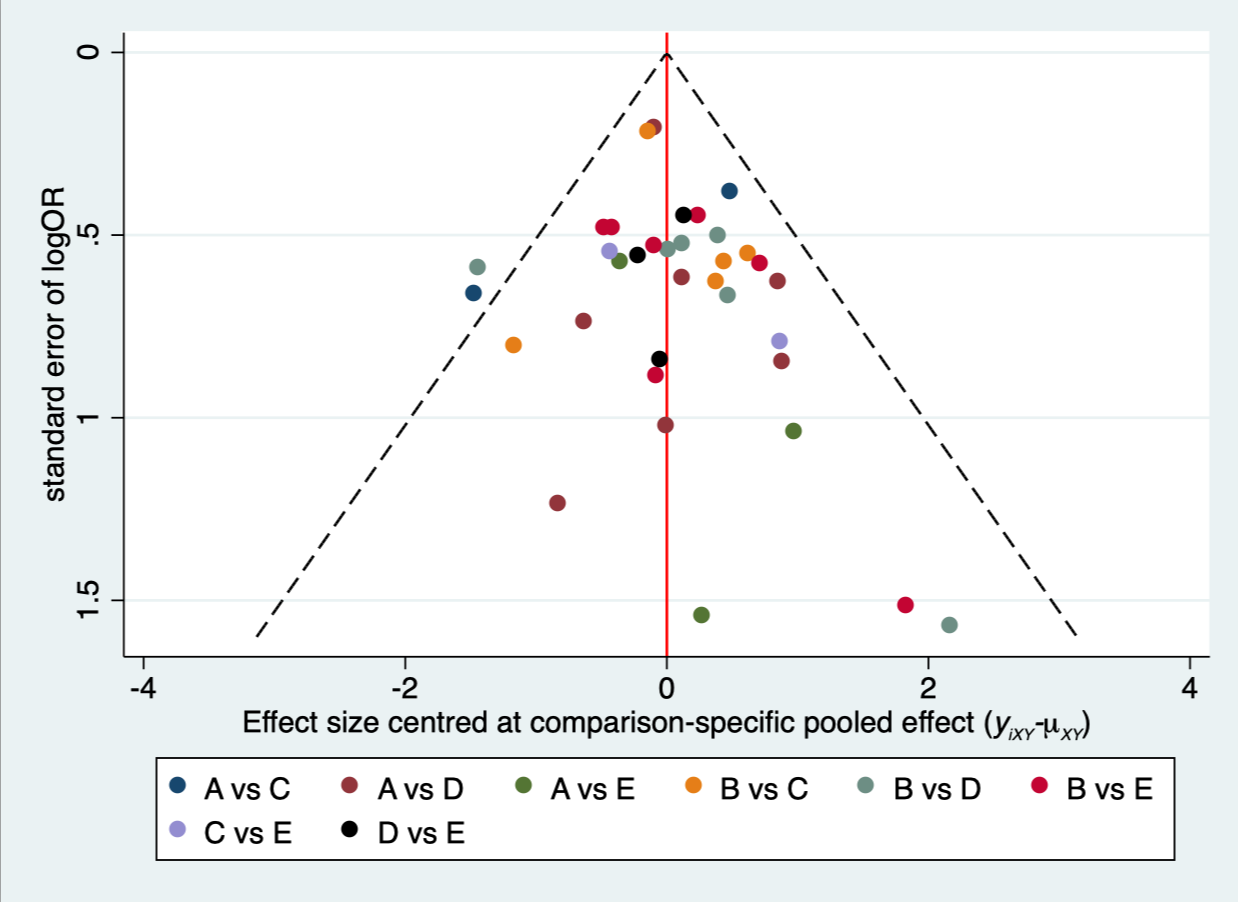

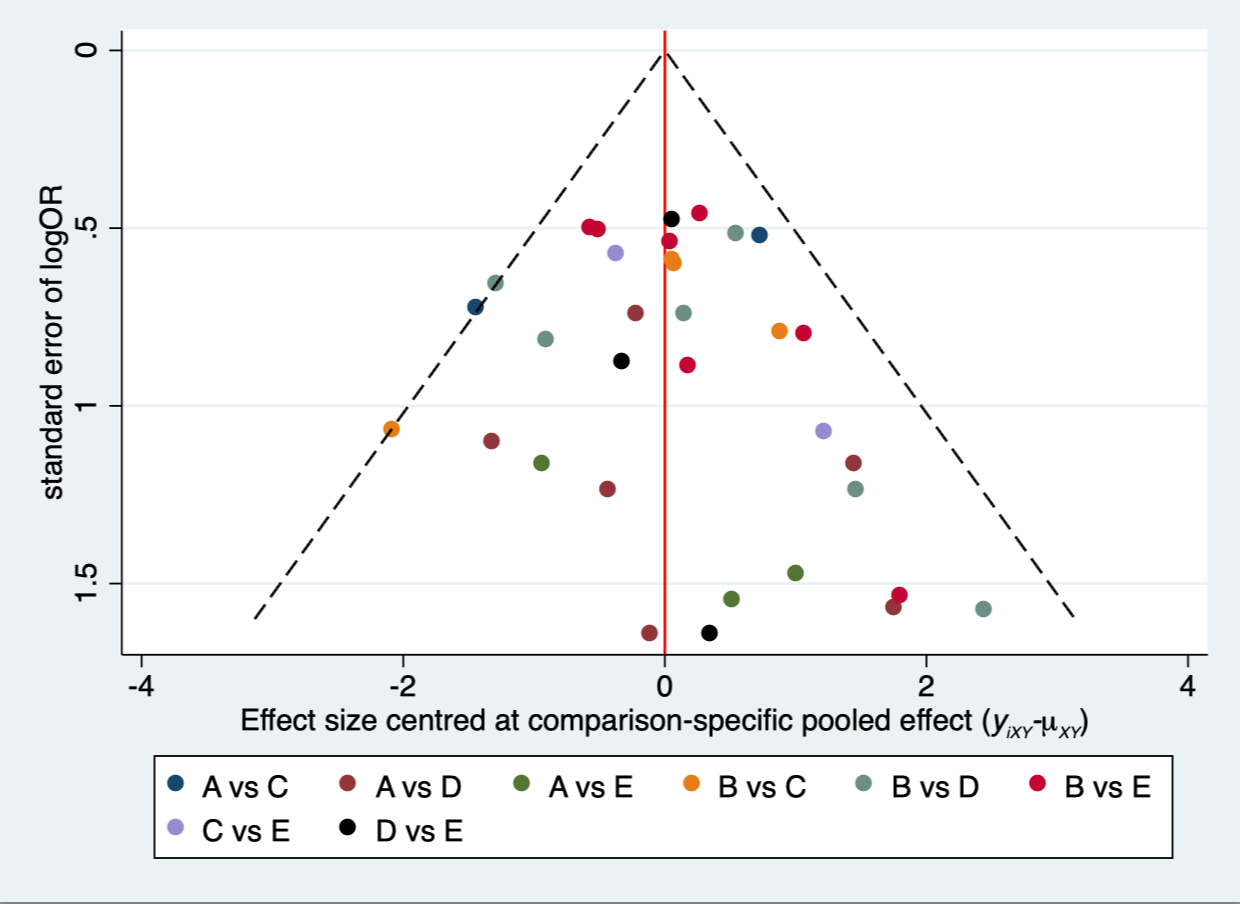


**C D**


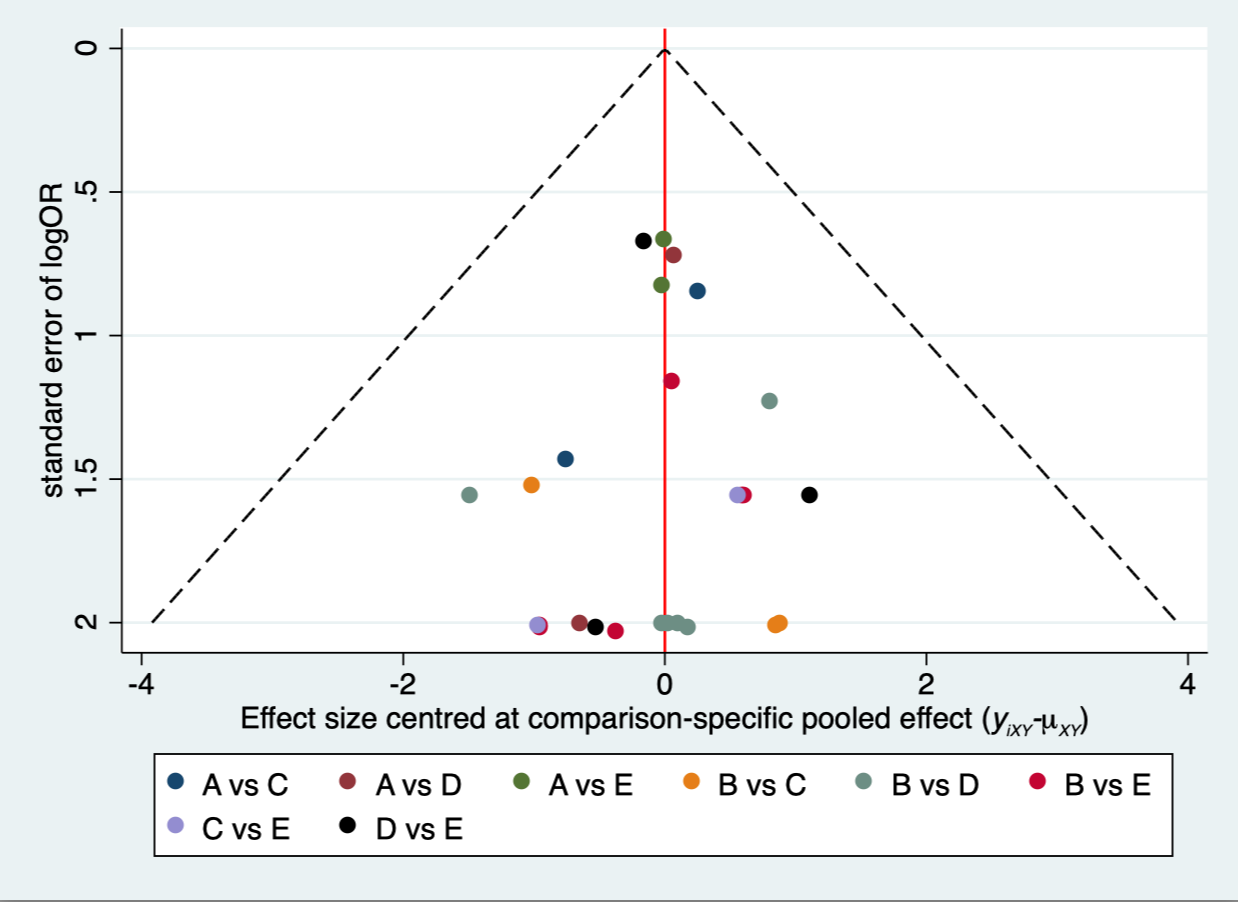

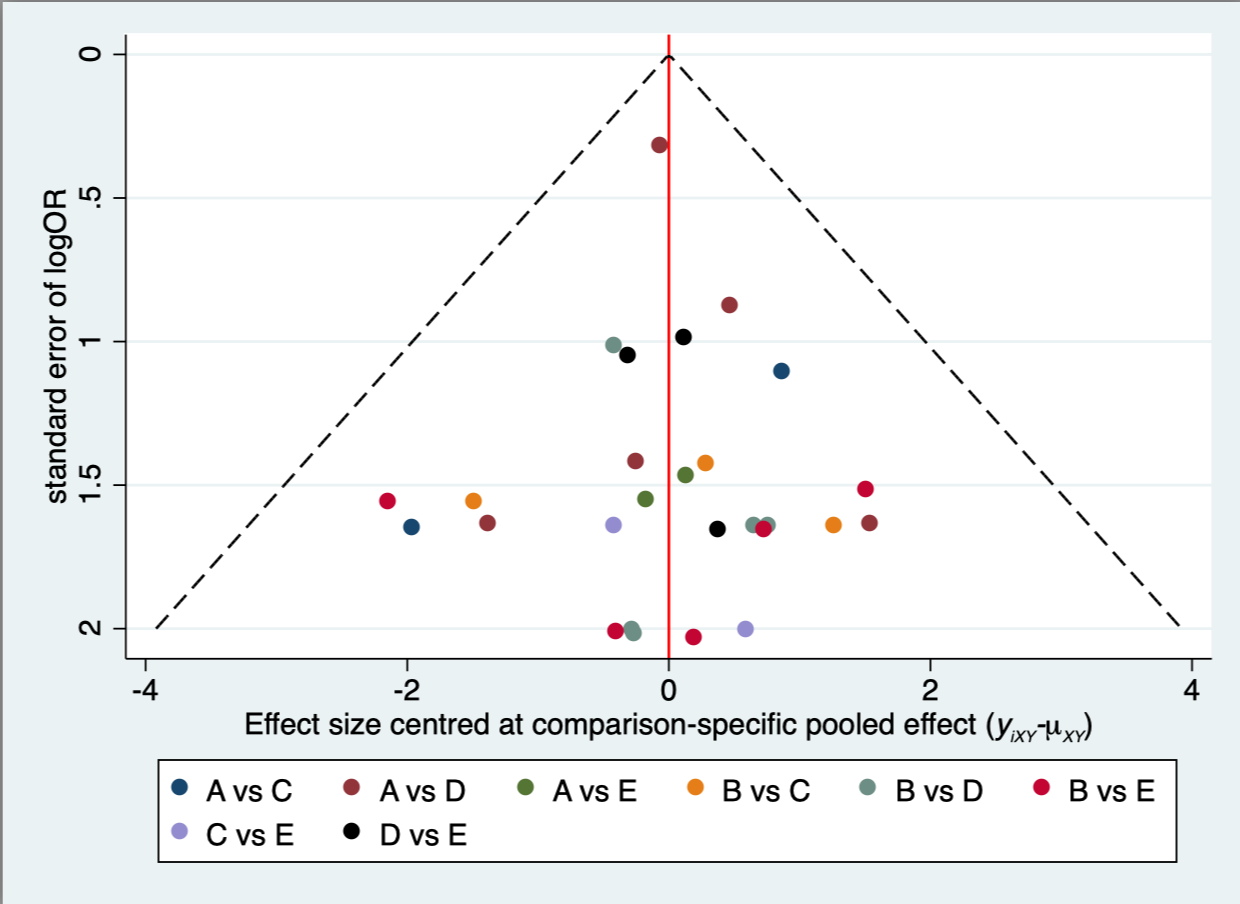


**E F**


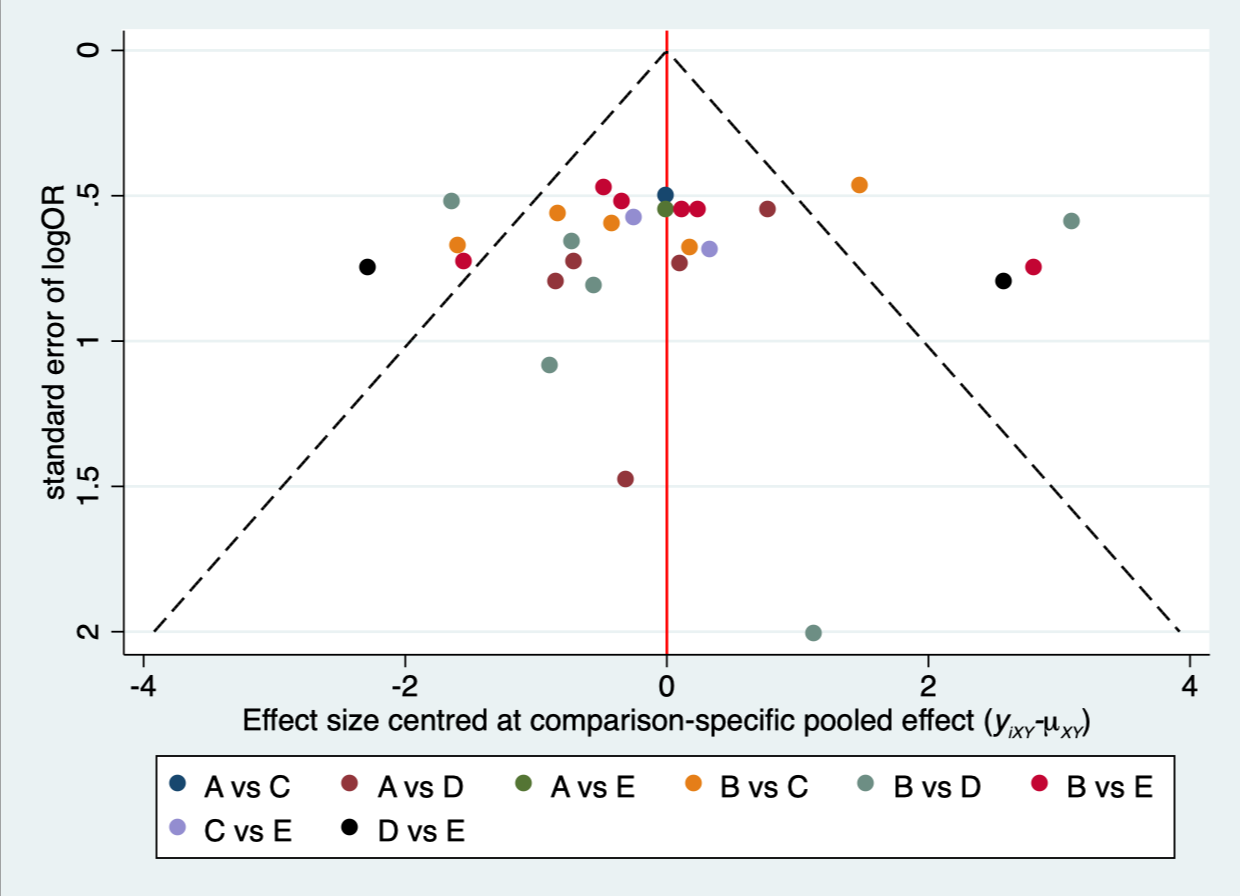

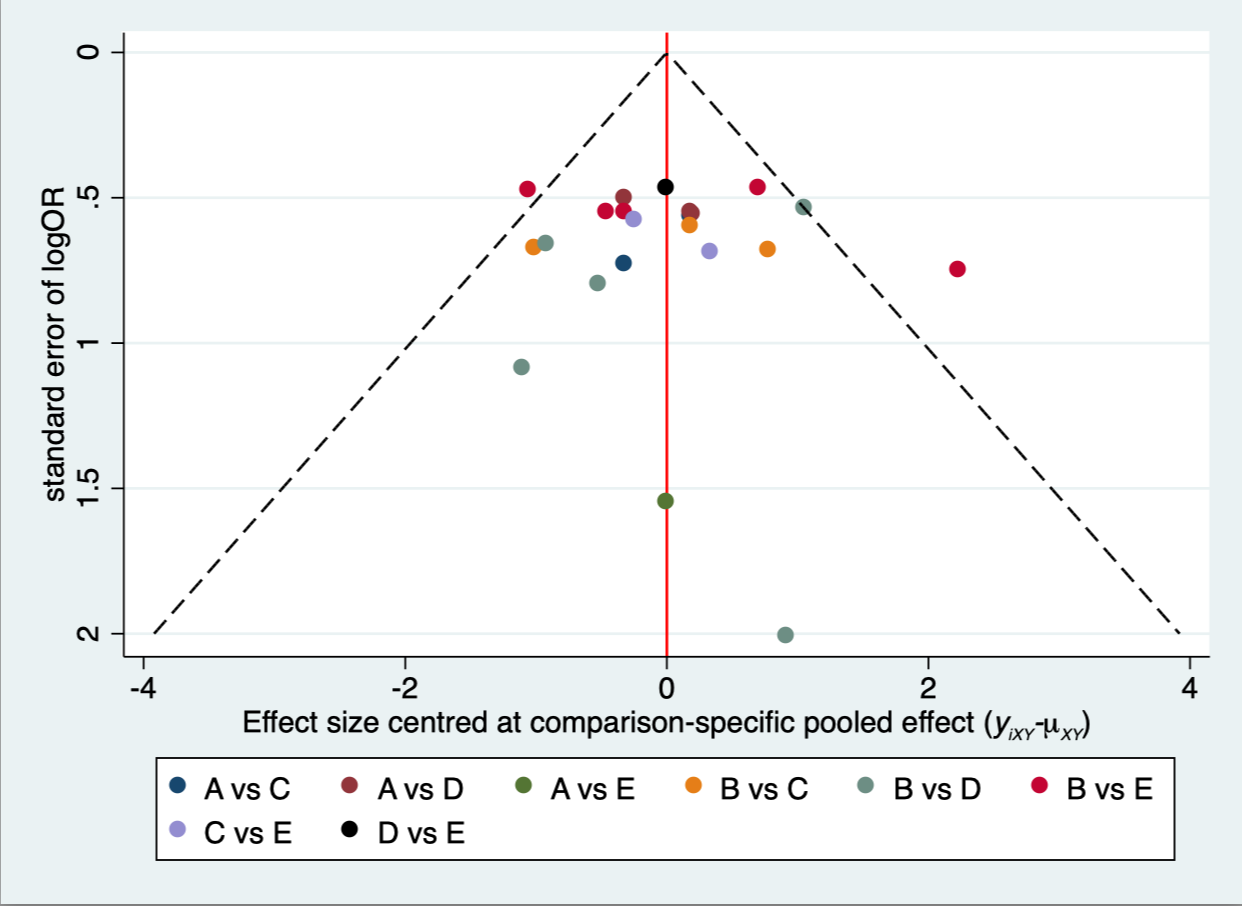


**Figure S1. Funnel plot of each outcome.** (A) MACE; (B) TLR; (C) all-cause death (D) myocardial infarction; (E) LLL; (E) BR.

The A to E in each funnel plot refers to five strategies (A: DCB only; B: DCB+BMS; C: First-generation DES; D: Second-generation DES; E: BMS).

**A B**


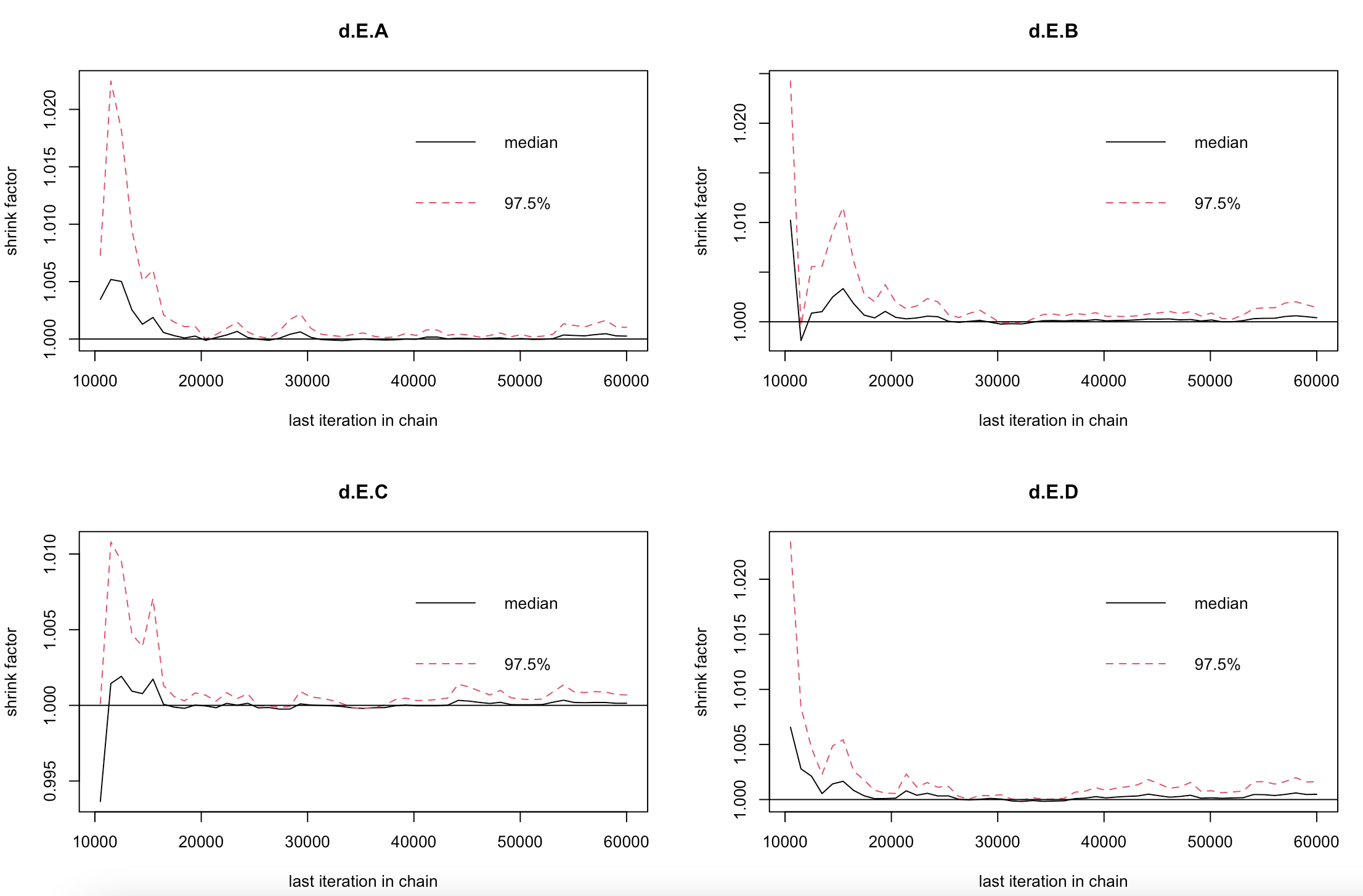

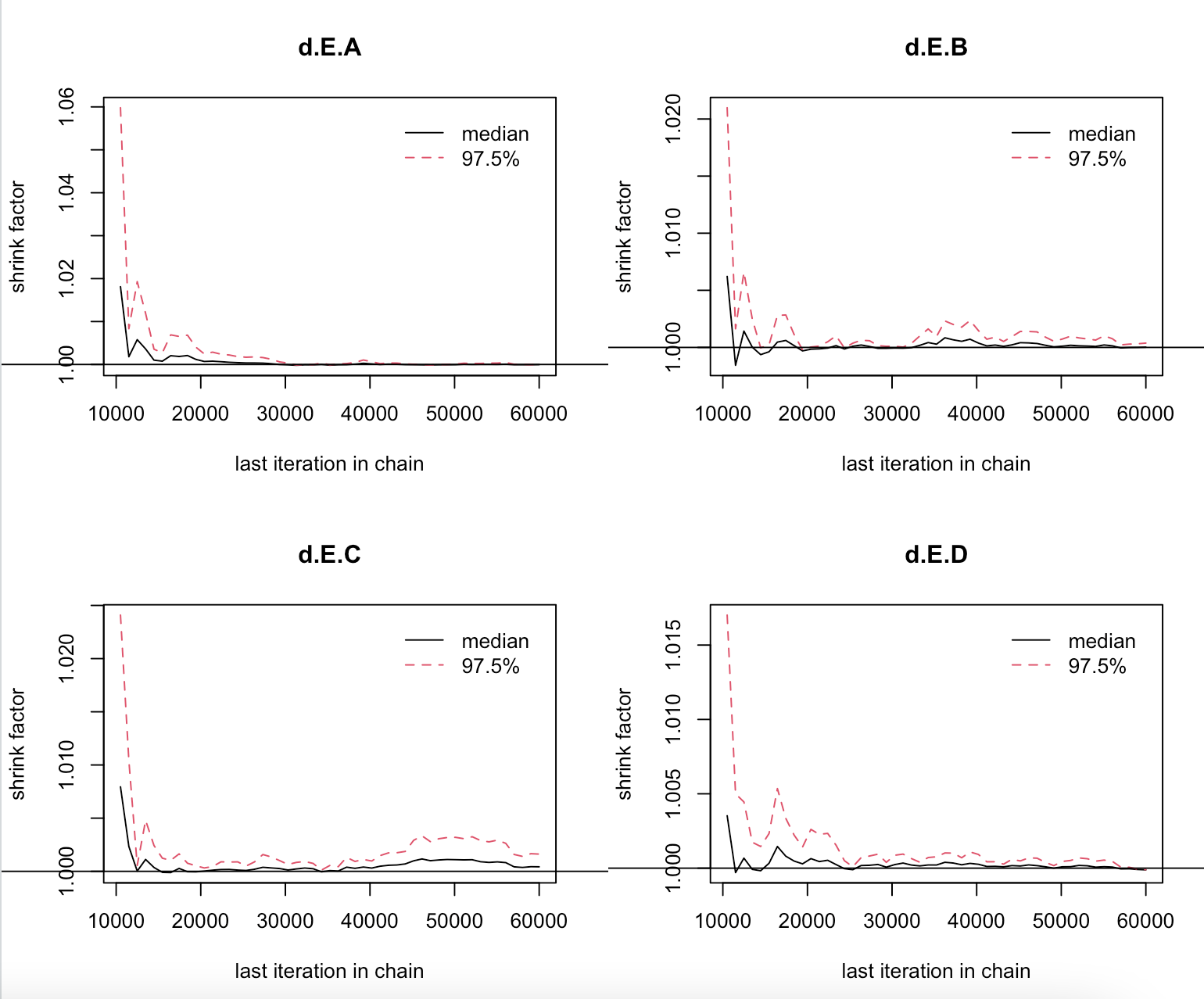


**C D**


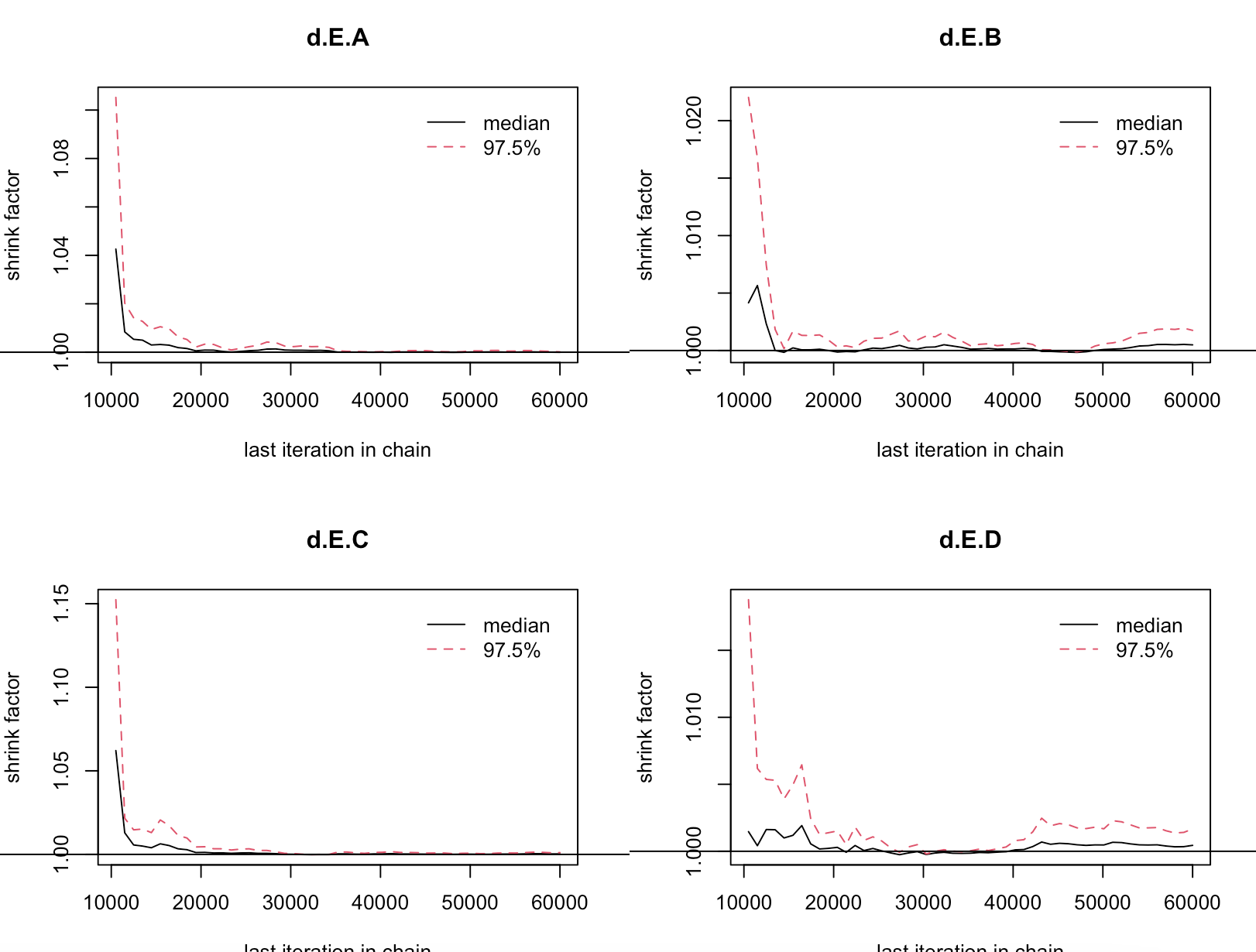

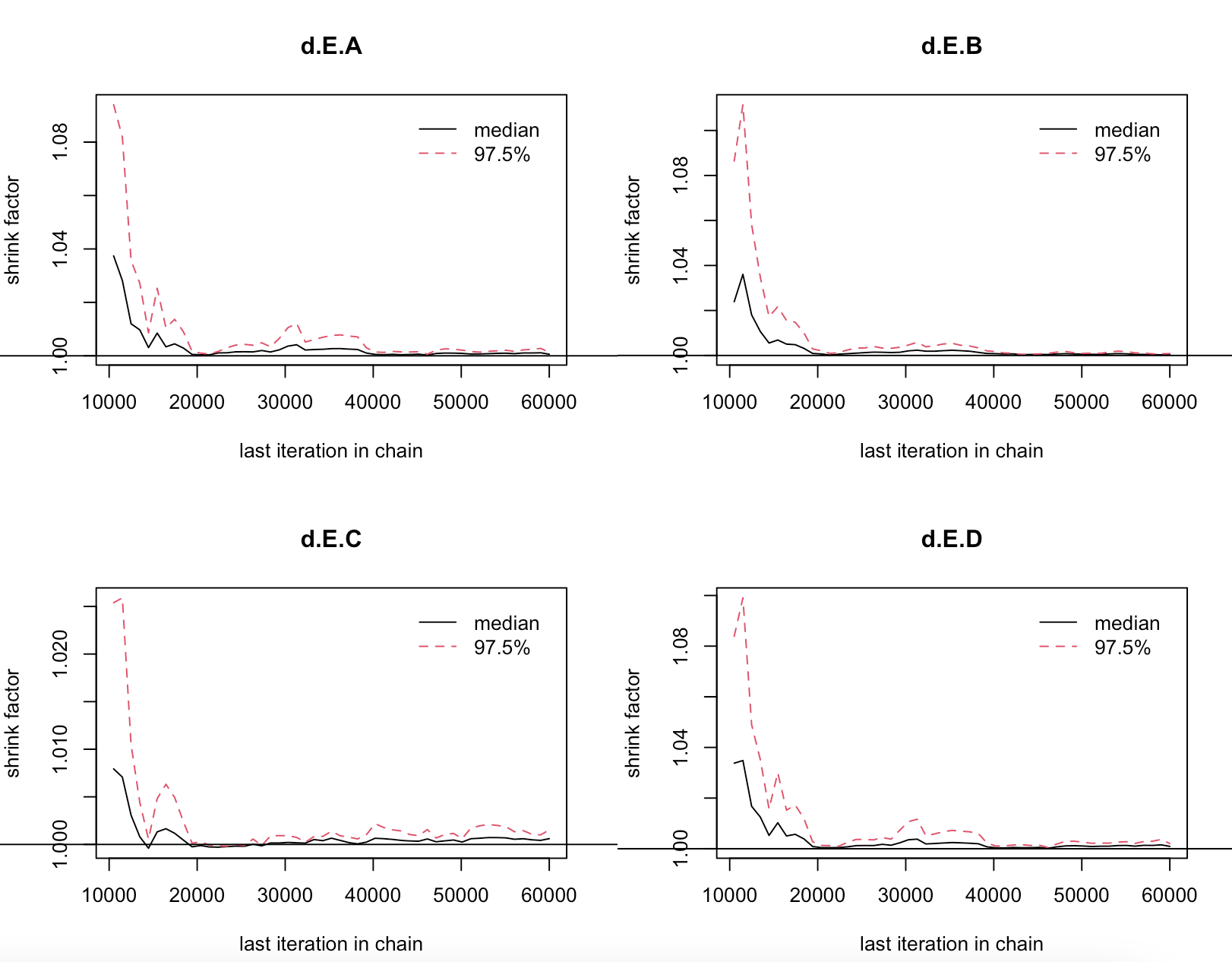


**E F**


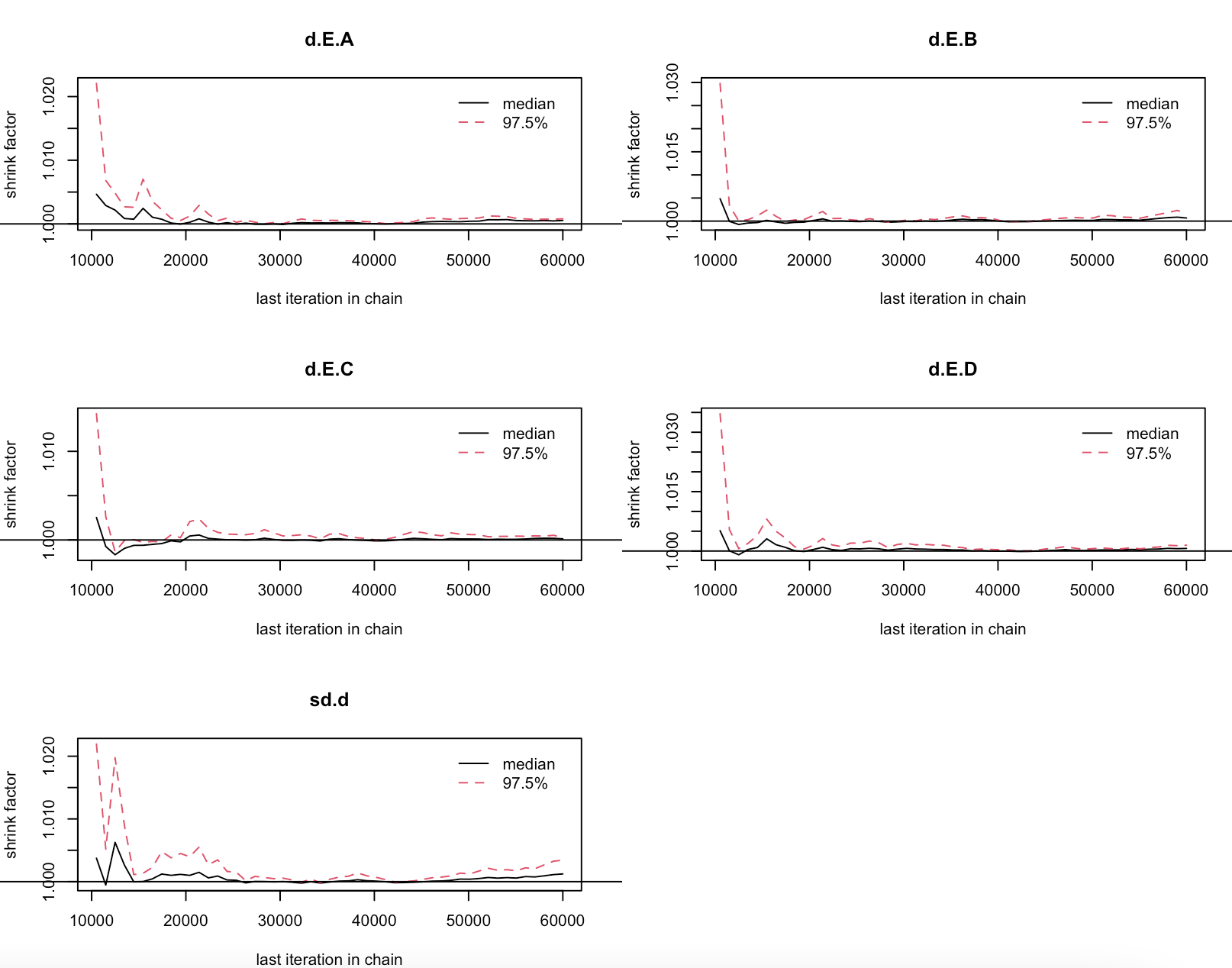

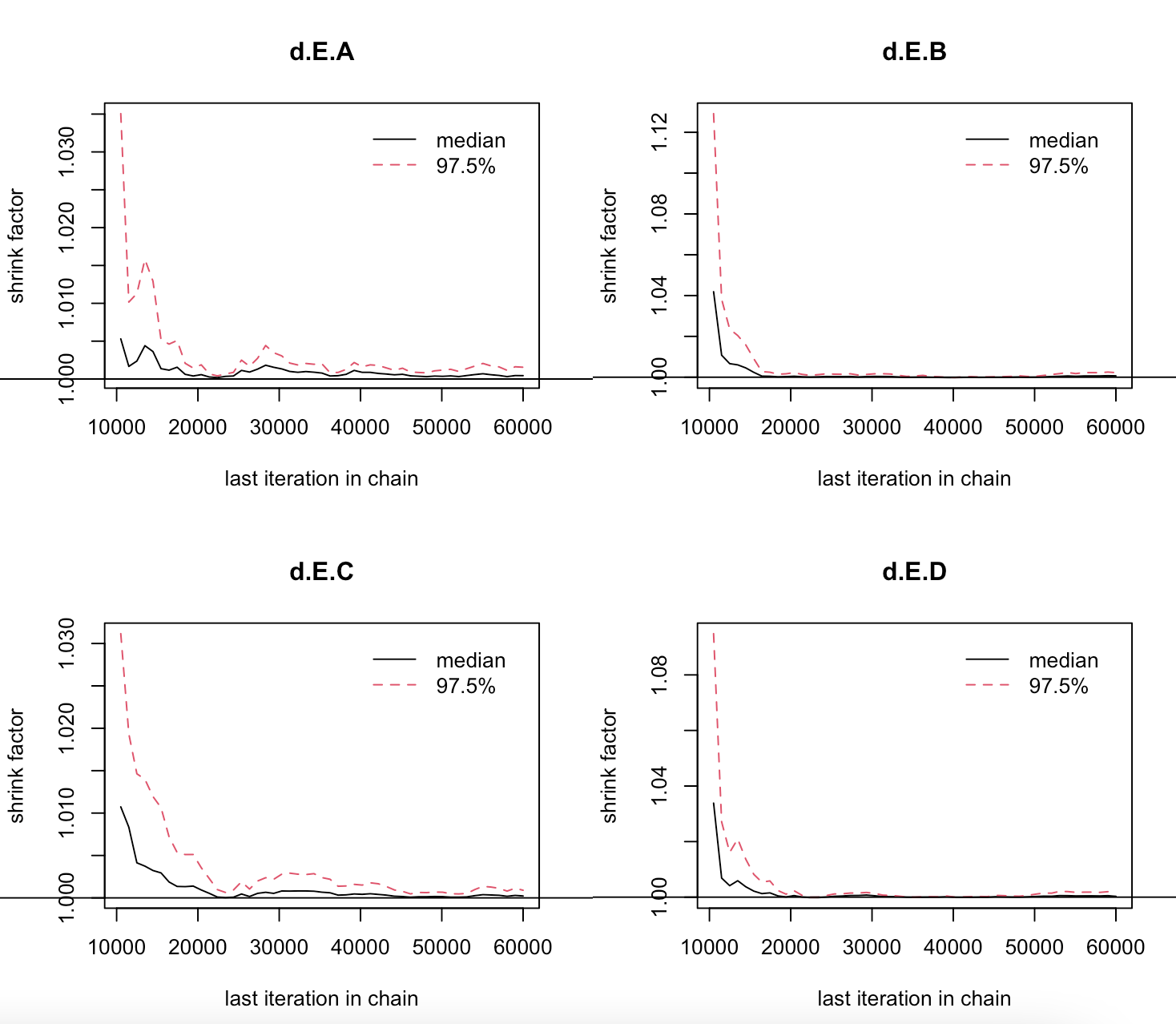


**Figure S2. Convergence plots of each outcome.** (A) MACE; (B) TLR; (C) all-cause death; (D) myocardial infraction; (E) LLL; (F) BR.

**A B**


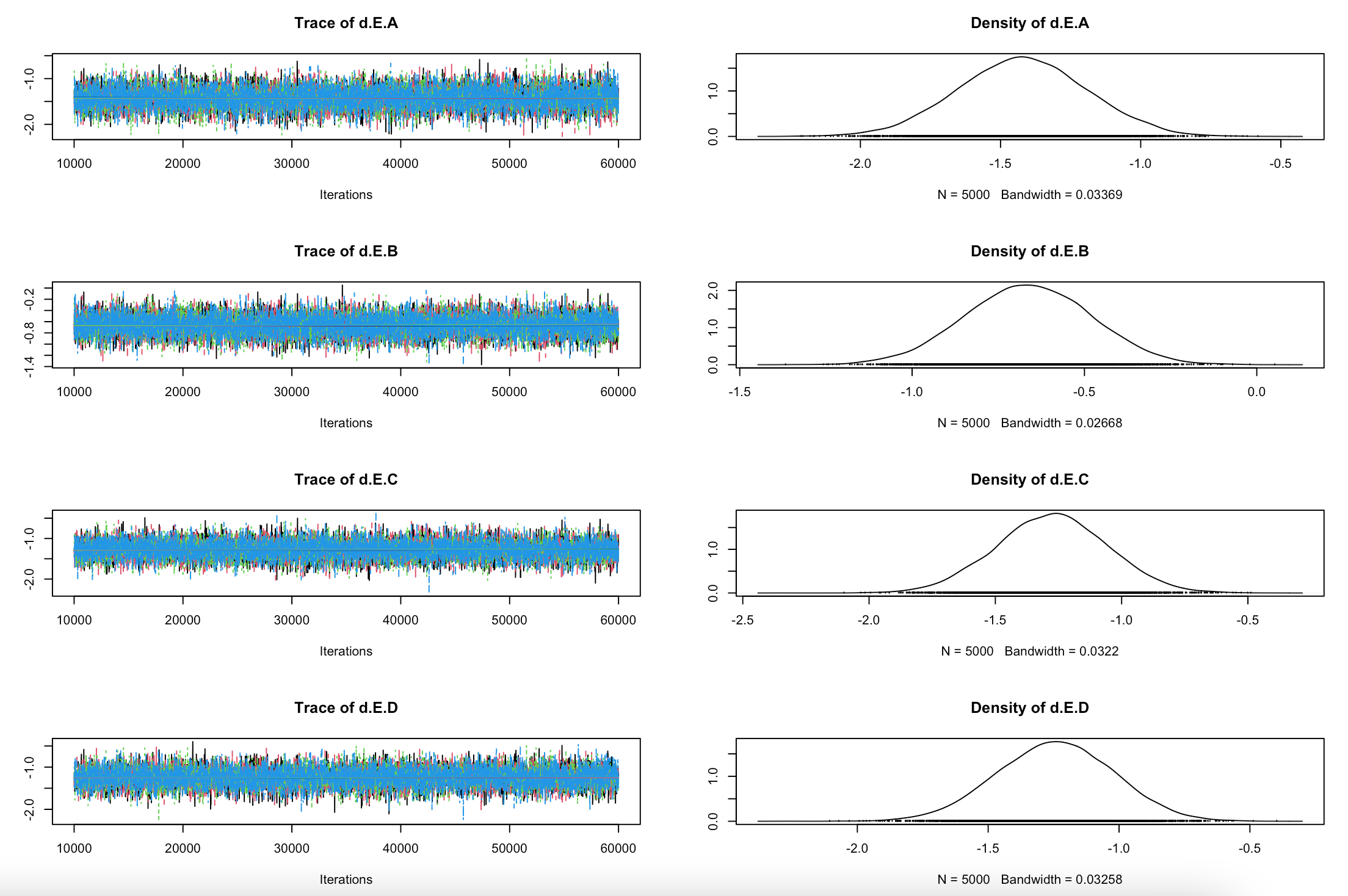

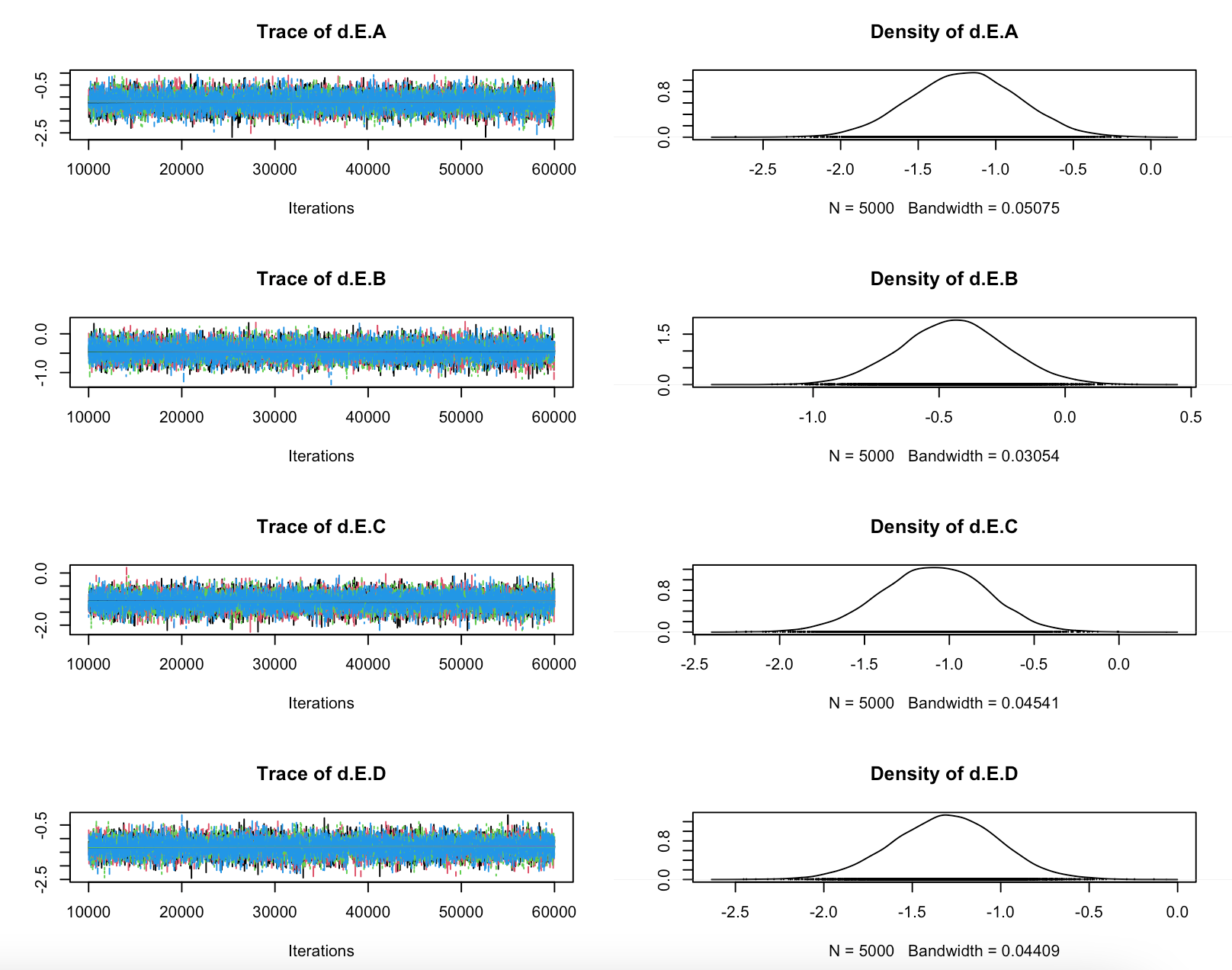


**C D**


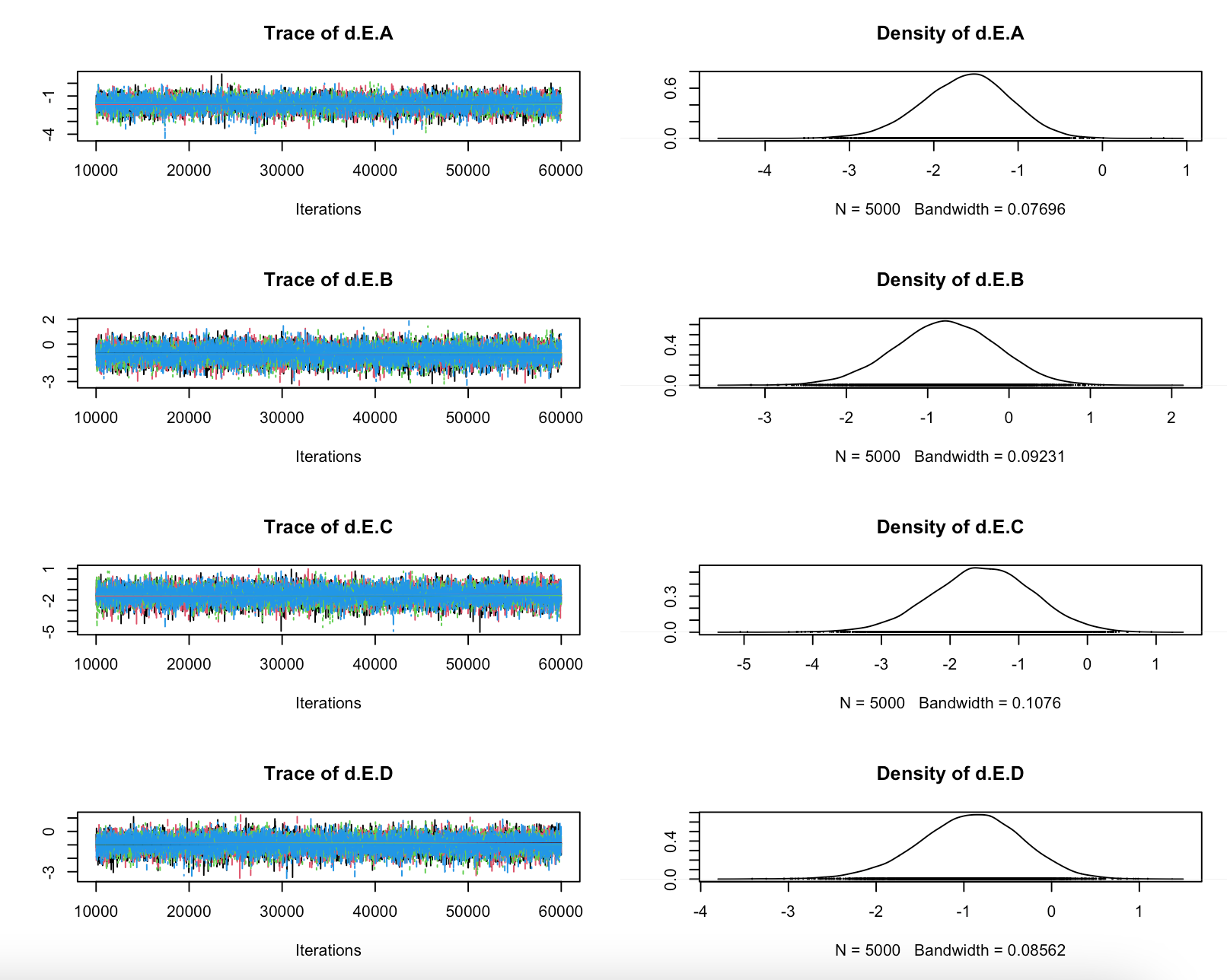

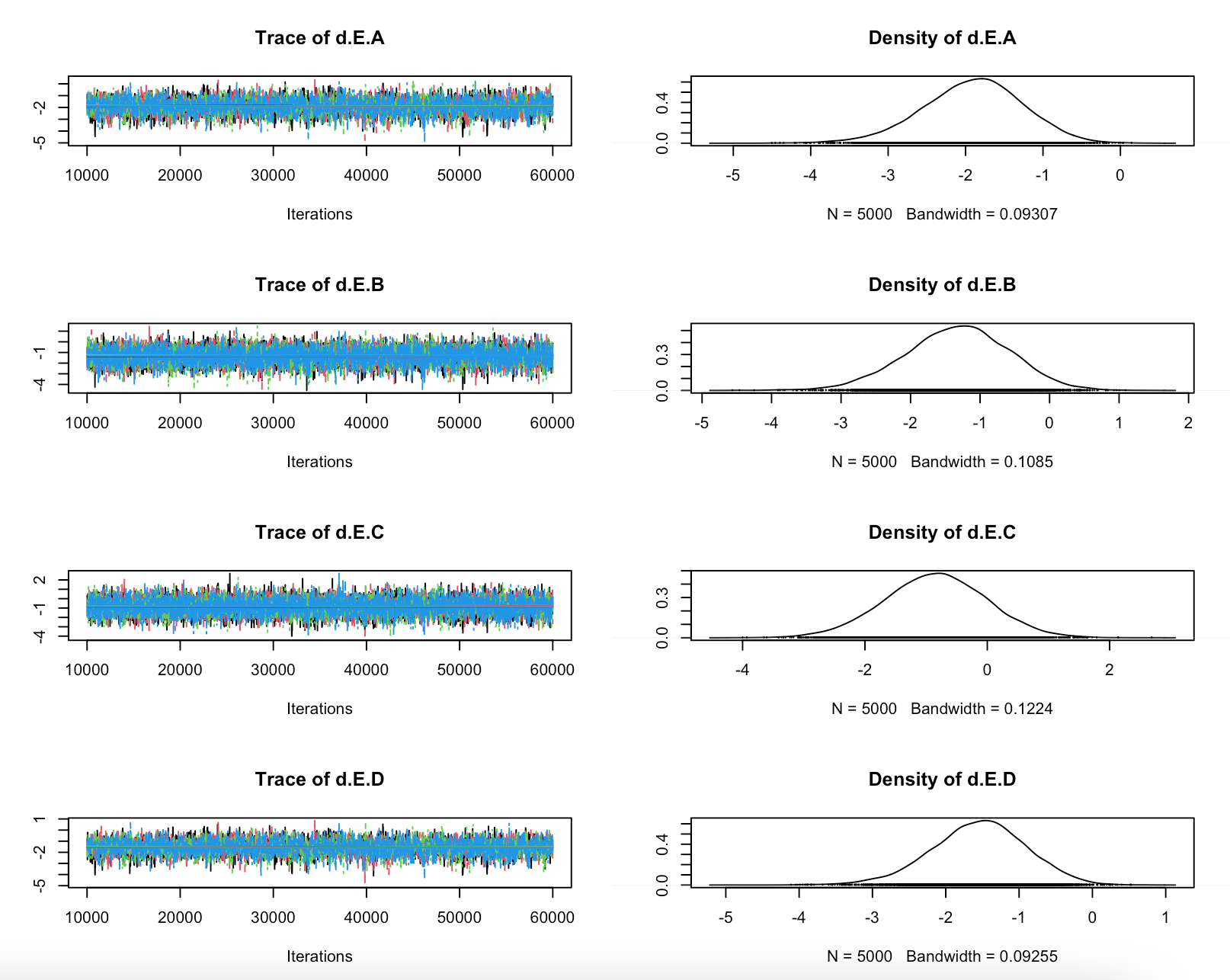


**E F**


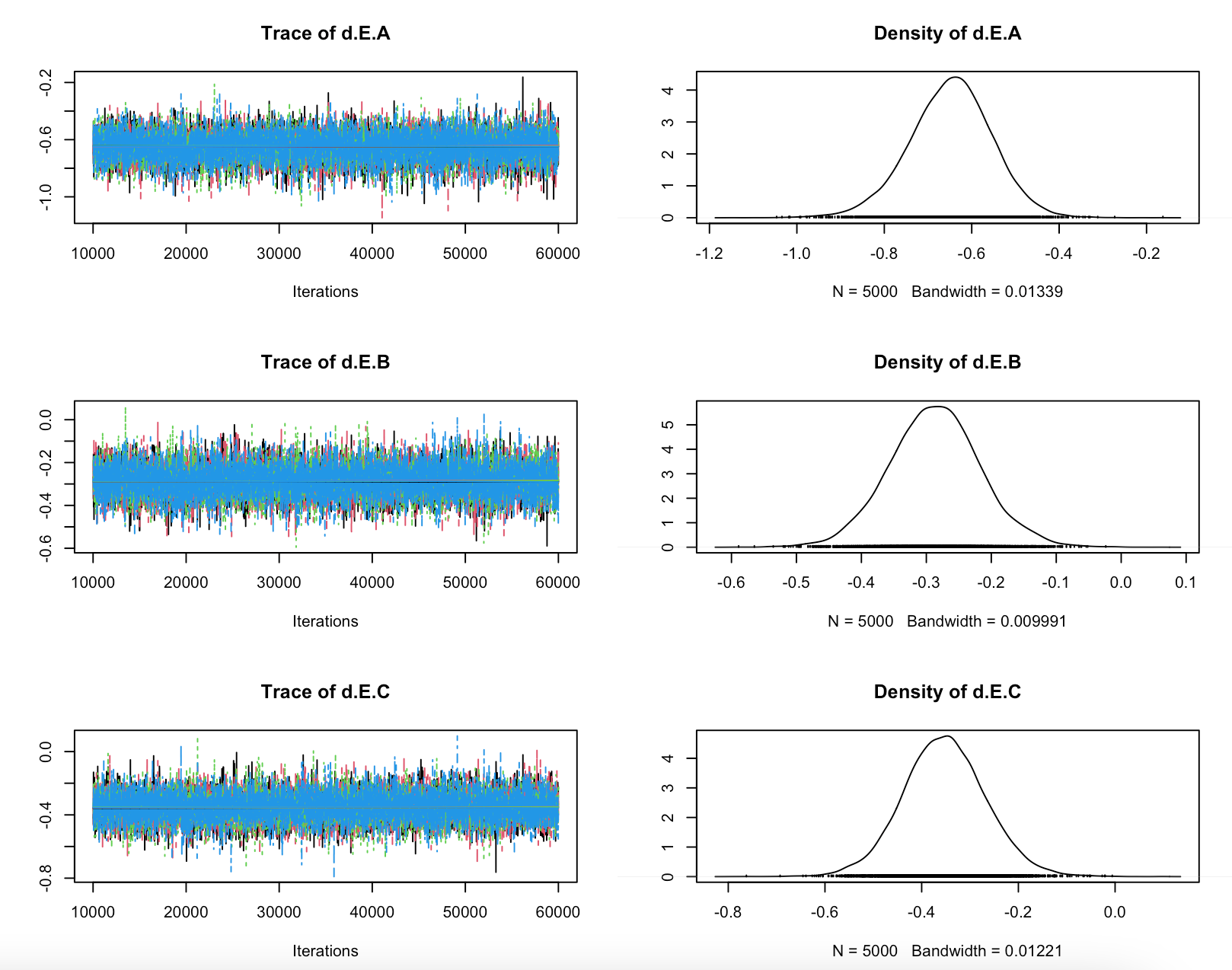

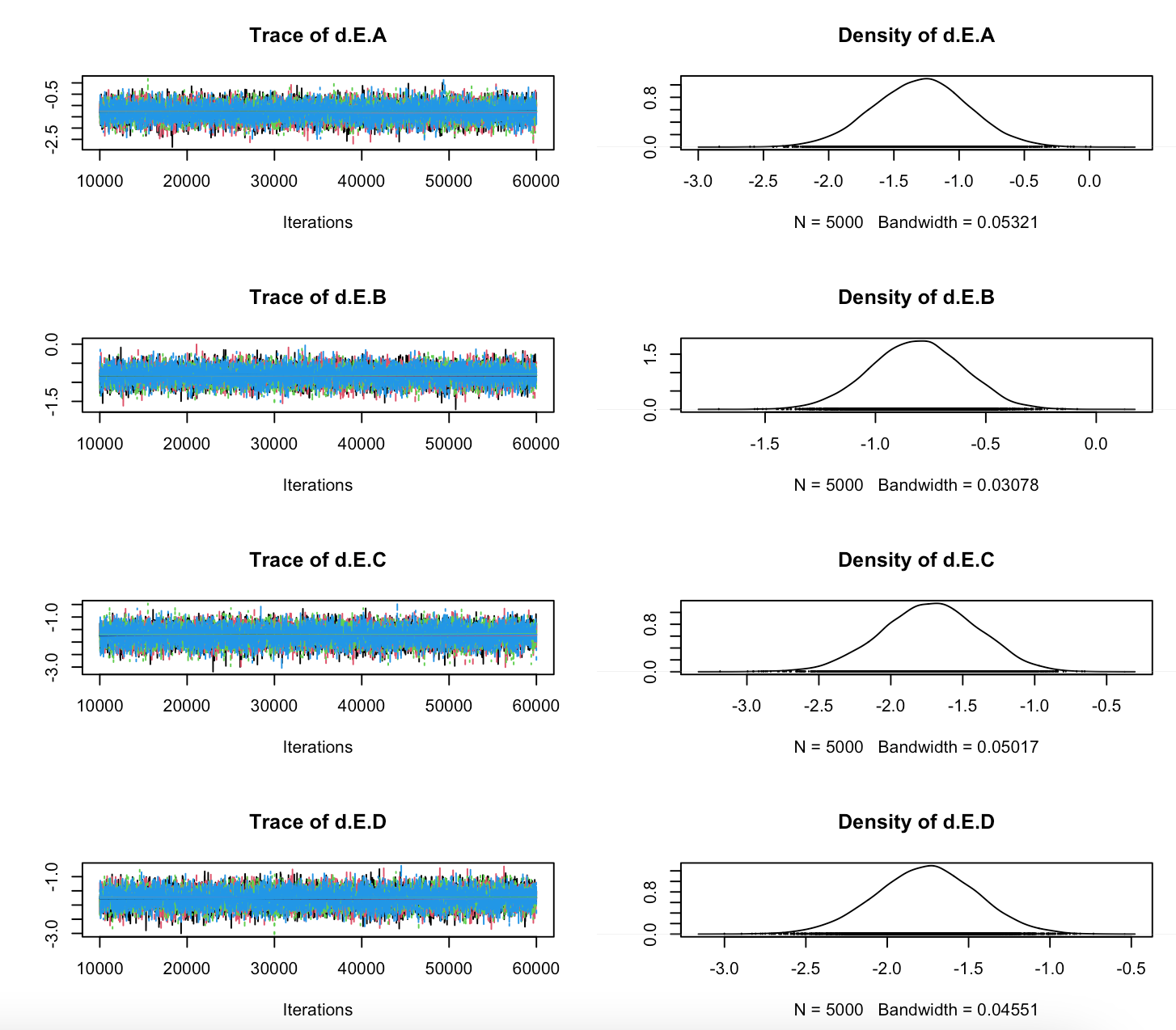


**Figure S3. Trace plots of each outcome.** (A) MACE; (B) TLR; (C) all-cause death; (D) myocardial infraction; (E) LLL; (F) BR.

If the trace plot (a graph visually showing the simulation result) has no specific pattern and the chains are entangled, it is considered that the convergence is good. The density plot is a posterior distribution (posterior density function) and if the shapes are significantly different for the same number of simulations, it means the data did not converge well.

**A B**


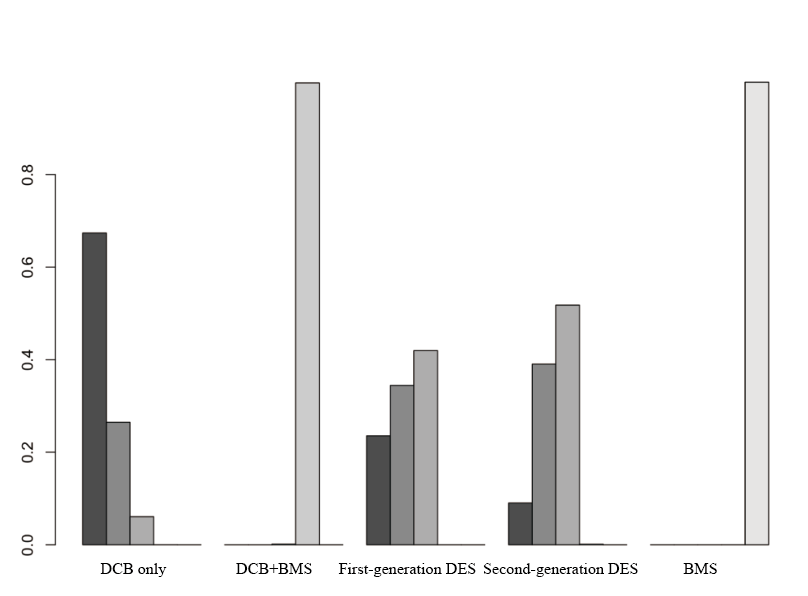

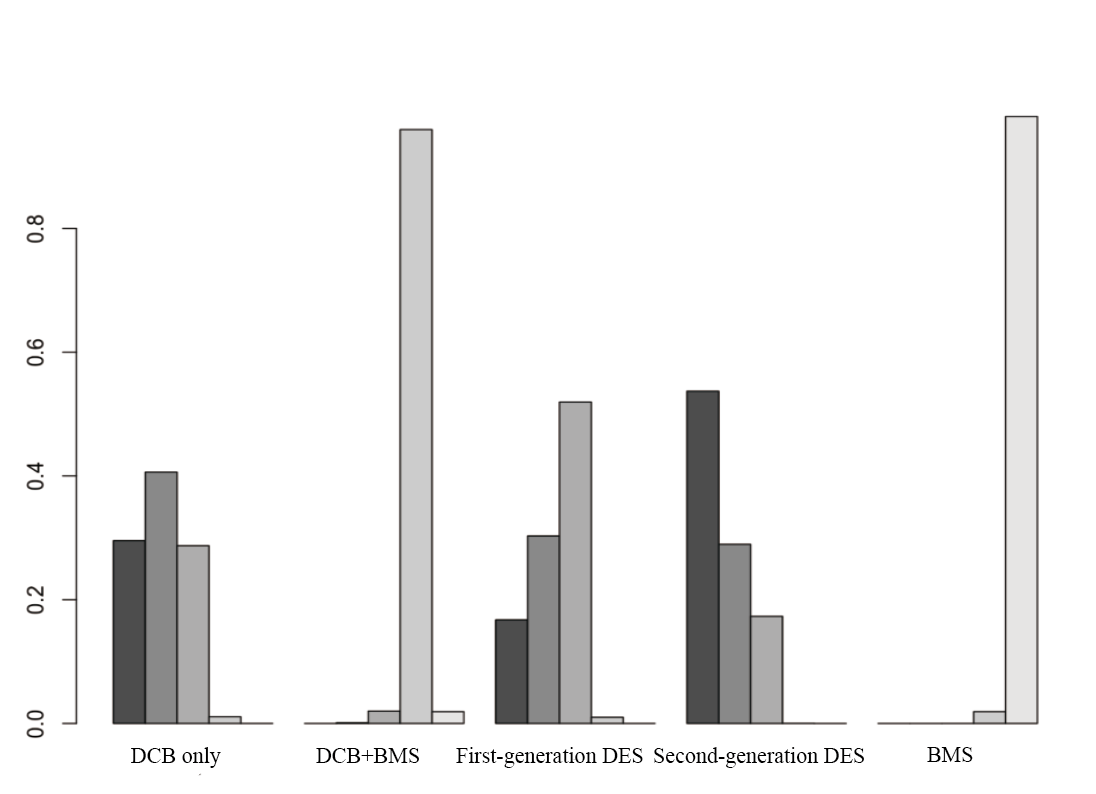


**C D**


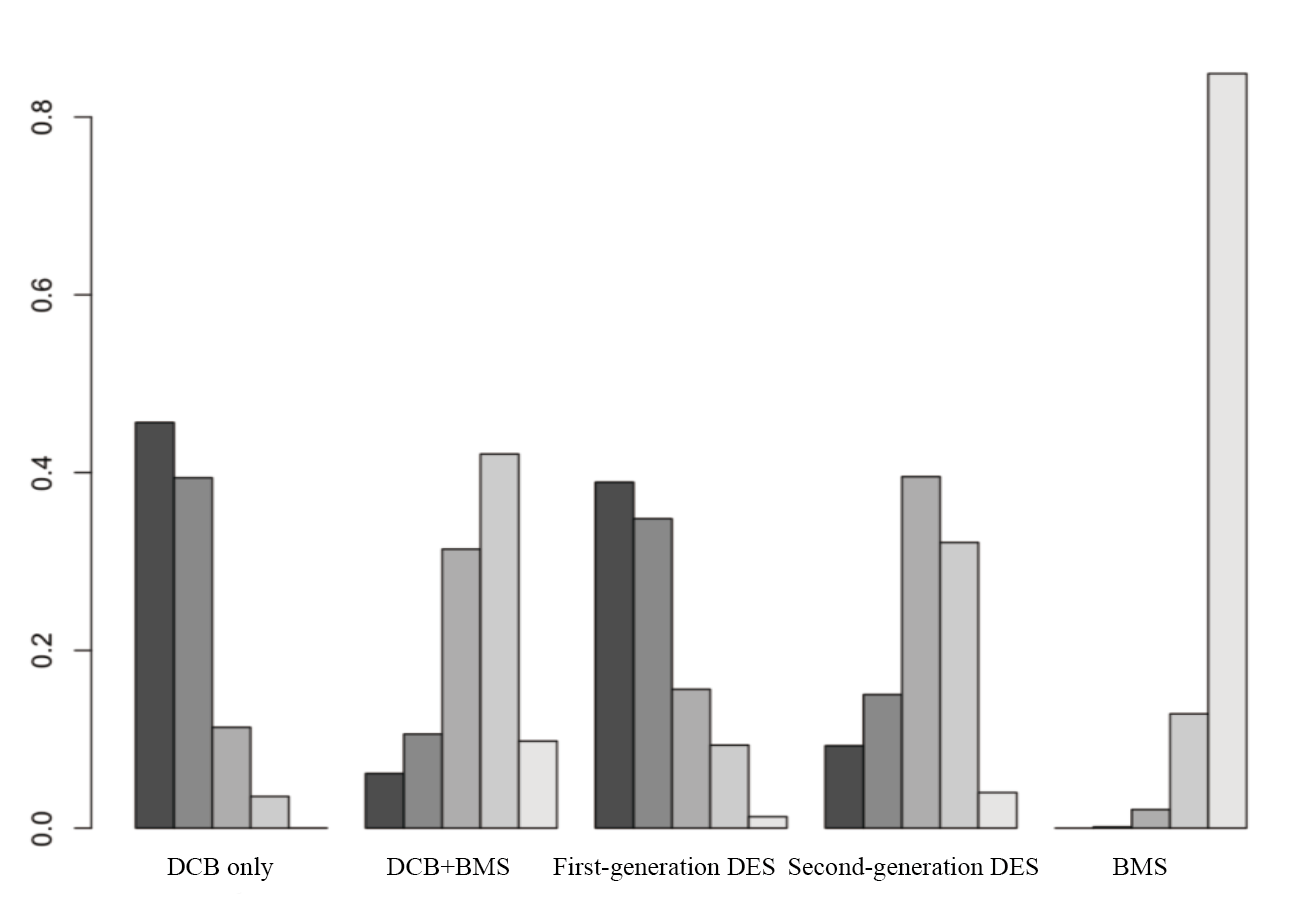

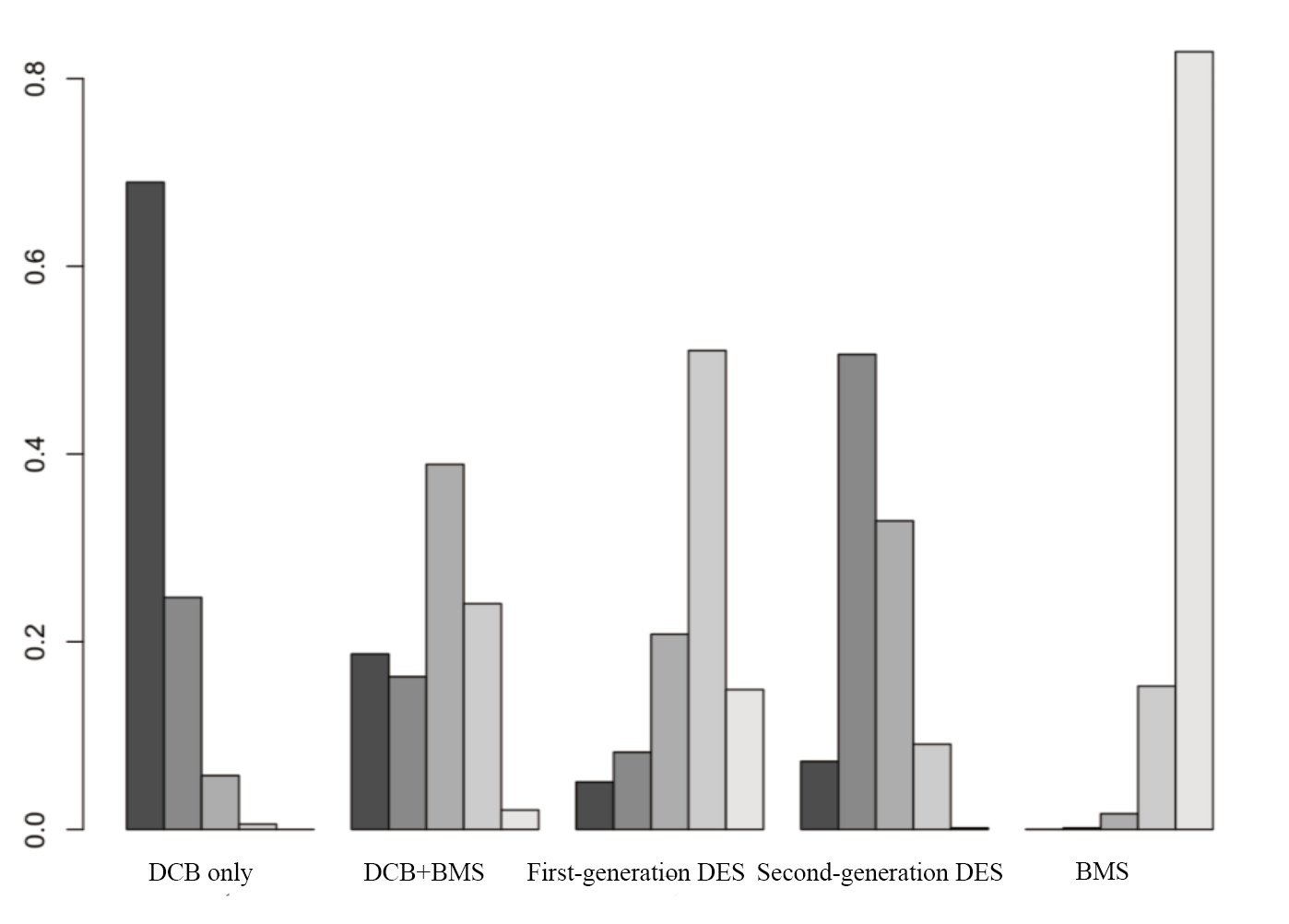


**E F**


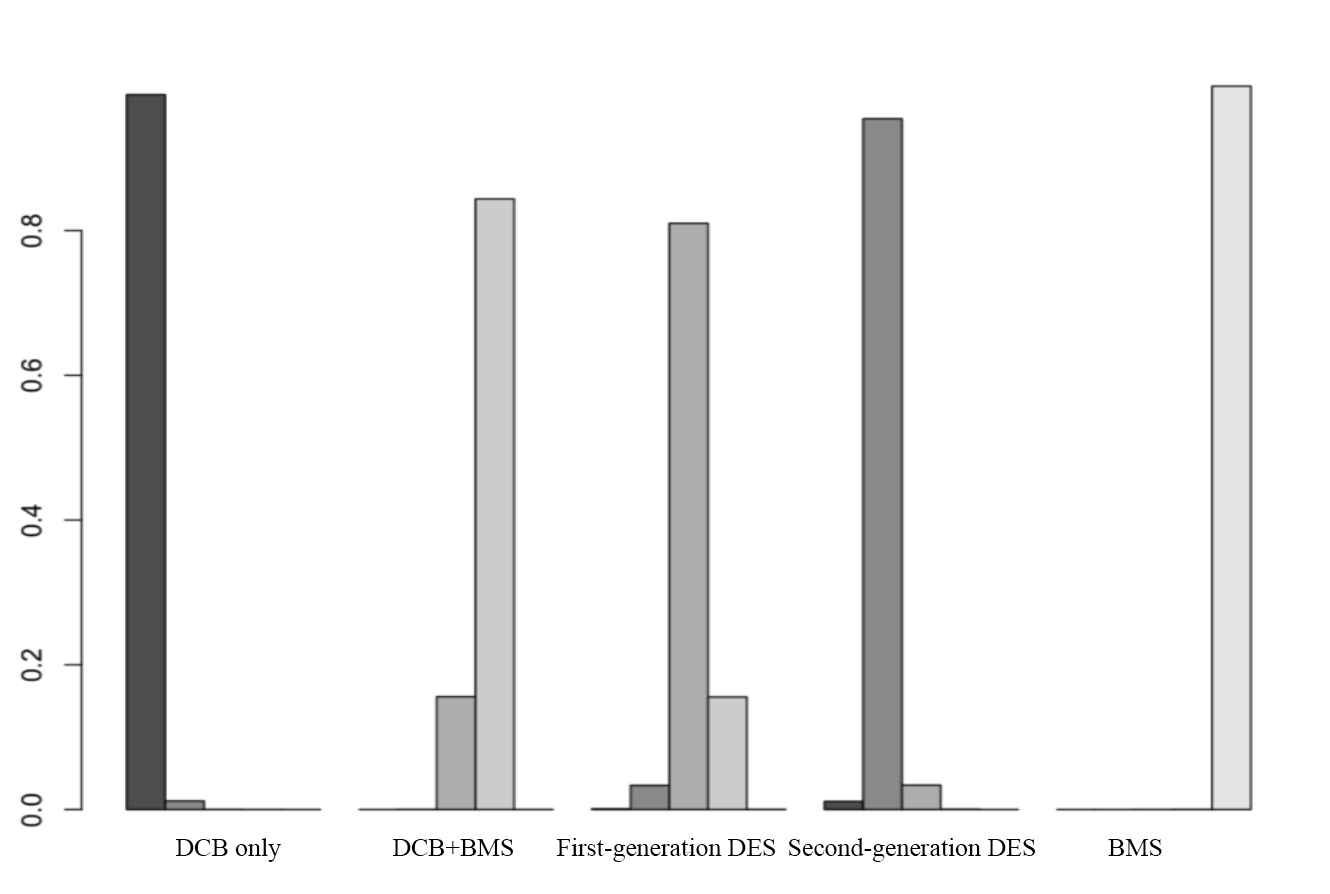

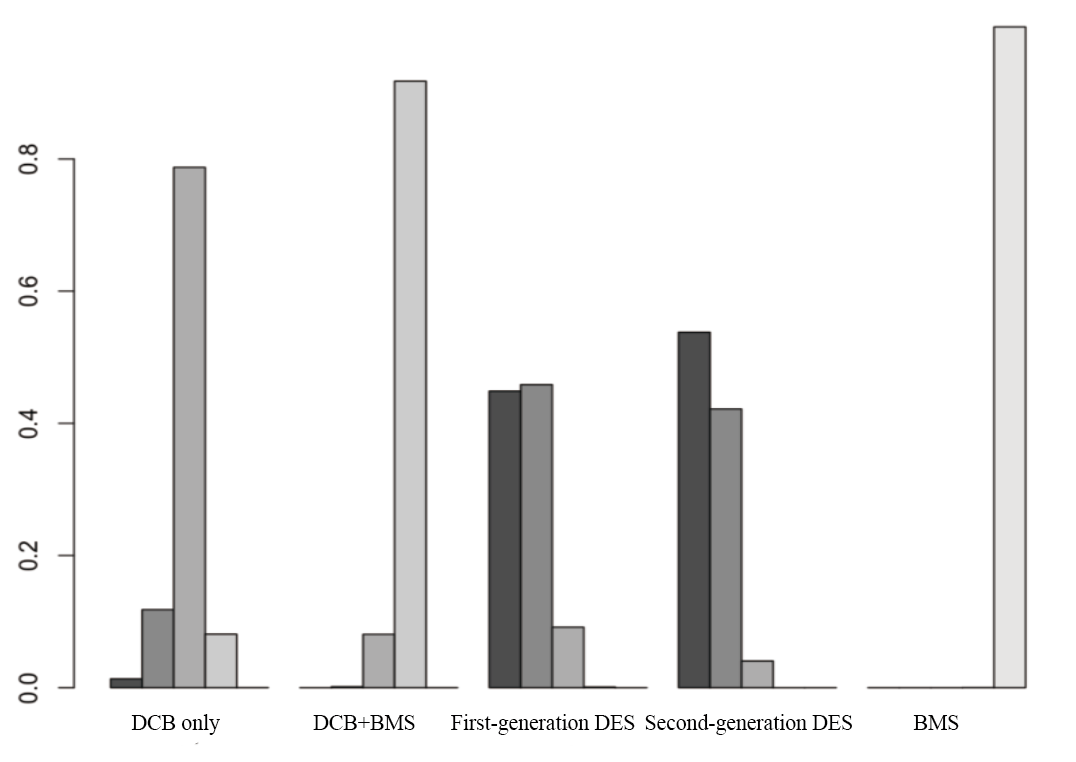


**Figure S4.** Rankograms on histogram of entire cohort. (A) MACE; (B) TLR; (C) all-cause death; (D) myocardial infraction; (E) LLL; (F) BR.

Figure S4A: DCB only is the optimal strategy for reducing the incidence of MACE (Probability of rank, 67%). First-generation or second-generation DES strategies rank second to third. DCB+BMS ranks fourth in five strategies (Probability of rank, 100%). BMS is the worst strategy in reducing MACE (Probability of rank, 100%). Supplementary Figure S4B: Second-generation DES ranks the optimal strategy in TLR (Probability of rank, 54%). DCB only and first-generation DES rank second to third. DCB+BMS and BMS rank fourth to fifth, respectively (Probability of rank is close to 100%). Supplementary Figure S4C: first-generation DES or DCB only are the optimal strategy for reducing all-cause death (Probability of rank, 40-46%). BMS is the worst strategy and the P value about 90%. Supplementary Figure S4D: DCB only ranks the optimal strategy for reducing myocardial infarction (Probability of rank, 69%), and BMS is the worst strategy (Probability of rank, 95%). Second-generation DES, first-generation DES and DCB+BMS rank second to fourth, respectively. Supplementary Figure S4E: DCB only, second-generation DES, first-generation DES, DCB+BMS and BMS rank from best to worst strategy for reduce LLL, respectively (Probability of rank, 80-99%). Supplementary Figure S4F: Second-generation DES and first-generation DES are the optimal strategies for reducing BR. In addition, DCB only ranks third in BR (Probability of rank, 78%), DCB+BMS and BMS are rank fourth to fifth (Probability of rank, 90-100%).
